# Polar Triptycene-Based Nonmetal Organic Frameworks Show Enhanced Hydrogen Adsorption

**DOI:** 10.1021/jacs.5c11317

**Published:** 2025-10-16

**Authors:** Megan O’Shaughnessy, Hang Qu, Xue Wang, Jacob B. Holmes, Lyndon Emsley, Joseph Glover, Roohollah Hafizi, Graeme M. Day, Andrew I. Cooper

**Affiliations:** 1 Department of Chemistry, 4591 University of Liverpool, Liverpool L69 7ZD, United Kingdom; 2 Institut des Sciences et Ingénierie Chimiques, École Polytechnique Fédérale de Lausanne (EPFL), Lausanne CH-1015, Switzerland; 3 National Centre for Computational Design and Discovery of Novel Materials MARVEL, École Polytechnique Fédérale de Lausanne (EPFL), Lausanne CH-1015, Switzerland; 4 School of Chemistry and Chemical Engineering, 7423 University of Southampton, Southampton SO17 1BJ, United Kingdom

## Abstract

Porous nonmetal organic frameworks (N-MOFs) are an emerging class of porous materials that can have desirable properties such as stability for iodine capture, proton conductivity, and xenon/ krypton separation. Here, we present the first experimental evidence of polymorphism in porous N-MOFs through the identification of two stable porous phases of a triptycene framework (T.Cl-α and T.Cl-β). We also report single-crystal structures for two isostructural porous N-MOFs (T.Cl-α and T.Br-α). All three polymorphs are porous to carbon dioxide and nitrogen, and T.Cl-α exhibits a remarkable hydrogen uptake of 7.2 mmol g^–1^ at 77 K and 1 bar. This hydrogen sorption far exceeds most other porous crystals and compares favorably with many MOFs under the same condition. The hydrogen uptake for T.Cl-α is anomalously high for its relatively modest pore volume (0.198 cm^3^ g^–1^ from N_2_ isotherms at 77 K), which we attribute to London-dispersion interactions and geometric confinement within subnanometer pores.

## Graphical abstract

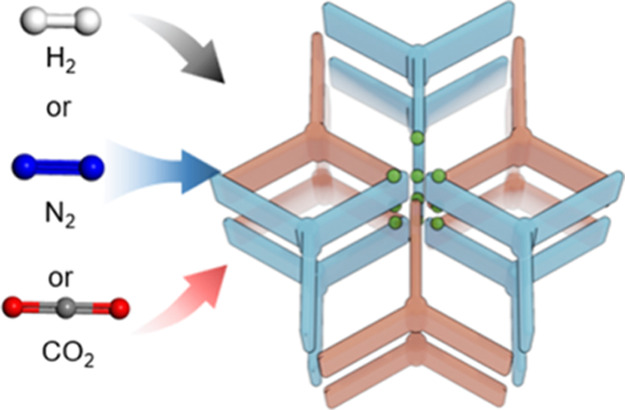

## Introduction

Porous materials are important for applications such as gas sorption, catalysis, and chemical separations.^1–3^ The exploration of porous solids with different chemical environments in the pores can lead to new physical properties. This has prompted researchers to study crystalline porous organic salt (CPOS) materials^4,5^ and nonmetal organic frameworks (N-MOFs).^6,7^ N-MOFs and CPOS materials are ionic porous molecular crystals that are characterized by noncovalent intermolecular interactions (Figure 1).^6,8^ These interactions are weaker than the strong directional bonds in metal–organic frameworks (MOFs)^1^ and covalent organic frameworks (COFs).^3^ This weak bonding ought to lead to some of the advantages observed in HOFs,^9^ such as allowing the materials to be solution processable. However, the more labile intermolecular interactions in porous molecular crystals can also have disadvantages. Specifically, it can lead to poor stability and, often, the loss of porosity when the solvent is removed from the pores.^10,11^ This can be a particular challenge for porous molecular salts, which tend to exhibit strong interactions with coordinating solvents in the pore channels. CPOS materials are therefore especially prone to pore collapse upon desolvation.^4,5^

**Figure 1:**
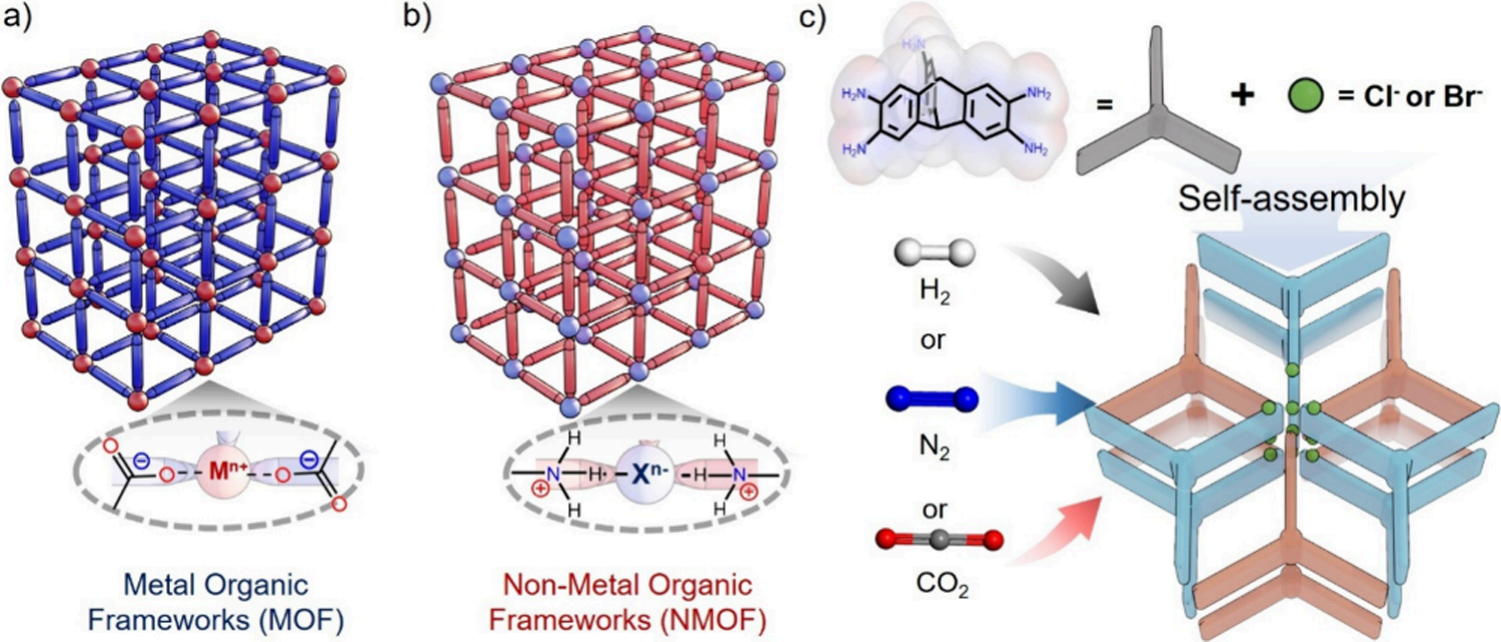
Inverse reticular design strategy (*c.f.*, Mottilo and Friščić for MOFs in *Chem. Commun.* **2015**, 8924) for porous nonmetal organic frameworks. a) MOFs comprise positively charged metal nodes (M^n+^) and negatively charged organic ligands (L^m–^). b) An inverse strategy is to design nonmetal organic frameworks with negatively charged nodes (X^n–^) and positively charged organic linkers (L^m+^). c) Design strategy for triptycene-based nonmetal organic frameworks.

Recently, we tackled this stability problem for porous salts by using crystal structure prediction (CSP) to predict N-MOFs where the global thermodynamic minimum crystal packing was porous.^6^ In principle, such materials should be stable to desolvation, at least if they remain crystalline, because there are no stable, dense crystalline forms available. We showed that these computationally designed N-MOFs were stable to solvent activation and to multiple iodine adsorption/desorption cycles,^6^ as well as being stable in water as proton conducting materials.^7^ However, these materials are exceptions to the rule: most CPOS materials show limited porosity or lose porosity upon solvent activation.^4,12,13^ Even our stable isoreticular N-MOFs^6^ showed relatively modest gas uptakes compared to more established porous solids.^1–3,14,15^ At first glance, therefore, the CPOS materials and N-MOFs reported so far would seem to be unpromising platforms for storing large quantities of gases, notwithstanding demonstrations of separation efficiency in these materials.^16^ However, the highly charged pores in N-MOFs present an unusual chemical environment, and we showed recently that this can translate to high proton conductivities.^7^ Likewise, pore channels that are lined with charged salts might interact strongly with gases. Specifically, these polar pores might adsorb weakly interacting gases, such as hydrogen, where gas-sorbate interaction energies define gas storage capacities more than pore volumes at low to moderate gas pressures.^17^

Our previous N-MOFs were stable and showed permanent porosity along with cyclable iodine capture properties,^6^ but growing single crystals for diffraction proved difficult owing to the rapid precipitation of these salts, which are poorly soluble in most solvents. Here, we used an alternative synthetic approach that employed halide salts of a triptycene hexamine, which are highly soluble in alcohols. This allowed us to obtain single crystal structures for the resulting porous halide triptycene salts, which are the first single crystalline examples of desolvated isostructural N-MOFs. We also found that these salts can have more than one porous polymorphic phase, a concept that we invoked in our earlier study^6^ but did not demonstrate experimentally.

These N-MOFs show some of the highest CO_2_ uptakes reported for porous organic salts,^18,19^ along with Type I nitrogen sorption isotherms, which are commonplace for porous frameworks but generally not observed thus far for porous organic salts.^20–23^ Of particular interest is the high H_2_ sorption values for these materials, which far exceed other porous organic salts and are competitive with MOFs.

## Results and Discussion

### Synthesis of Stable Porous Triptycene N-MOFs

The amine building block used in this work, 2,3,6,7,14,15-hexaaminotriptycene (HATT), is unstable in air.^24^ However, chloride salts of HATT can be prepared directly from the stable intermediate, N2, N3, N6, N7, N14, N15-hexakis­(diphenylmethylene)­triptycene-2,3,6,7,14,15-hexaamine^25^ (SI Scheme 1c). Here, we extended this approach to bromides via an analogous procedure with HBr (SI scheme 1a). Both Cl (**T.Cl**) and Br (**T.Br**) salts were found to be only soluble in alcohols, but in those solvents they had far greater solubilities than our previously reported N-MOFs.^6,7^ We therefore used methanol (MeOH) as the good solvent and attempted crystallizations with various antisolvents (see SI). This yielded two porous polymorphs of **T.Cl** (**T.Cl-α** and **T.Cl-β**) and one porous phase of **T.Br** (**T.Br-α**), which was isostructural with **T.Cl-α** (Figure 2).

**Figure 2:**
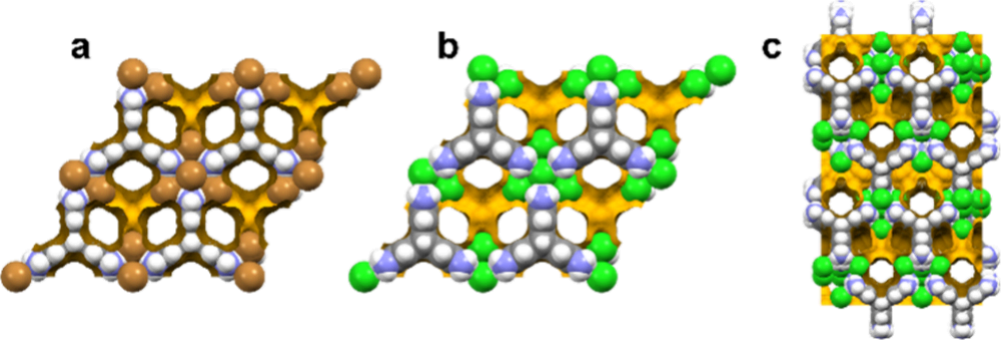
Space-filling representation of the crystal structures of a) **T.Br-α**, b) **T.Cl-α** and c) **T.Cl-β**

The first porous polymorph, **T.Cl-α**, was also reported by Xie et al. (HAT_Cl)^26^ during the preparation of this manuscript, but there it was studied in a different context (noble gas separation). We also obtained a second isostructural material, **T.Br-α** (Figure 2a).

Single crystals of **T.Cl/Br-α** were obtained from vapor diffusion crystallization with CHCl_3_ as the antisolvent. The structures crystallize in the trigonal space group, *P*3̅*m*1. As discussed in more detail below, only 4 of the 6 amines were found to be protonated, counter to statements in a related study.^26^ In the isoreticular crystal structures of T-Cl/Br-α the triptycene molecules are positioned around a 3-fold axis, which makes it difficult for X-ray diffraction (XRD) to clearly distinguish between NH_3_^+^ and NH_2_ groups. Therefore, positional disorder was used to model the occupancy of the nitrogen species, with NH_2_ assigned an occupancy of 2/3 and NH_3_^+^ assigned 1/3. Additionally, a Cl^–^ or Br^–^ anion is located within the one-dimensional channel along the 3-fold axis and also exhibits positional disorder.

We suggested previously that N-MOFs might have multiple porous polymorphs, based on CSP studies,^6^ but we did not present any experimental examples of this. Here, single crystals of a second porous polymorph of **T.Cl**, **T.Cl-β**, were grown via vapor diffusion over 2 days of THF into a solution of T.Cl in MeOH. X-ray diffraction analysis revealed that **T.Cl-β** had crystallized in the orthorhombic space group *Pnma* with pores oriented along the crystallographic *a* axis. Again, only four out of the six amines were protonated. In the single crystal structure of a solvate of **T-Cl-β** (Figure 5), MeOH molecules disrupt the symmetry of the packing arrangement, allowing the H_3_^+^N—C (1.47 Å) and NH_2_—C (1.41 Å) bonds to be distinguished based on their bond lengths. The distance between two adjacent NH_3_^+^ groups (2.91 Å) attached to the same aromatic ring is greater than that between an NH_3_^+^ and an NH_2_ group (2.86 Å), suggesting electrostatic repulsion between neighboring NH_3_^+^ groups. The formation of hydrogen bonds between NH_2_ and NH_3_^+^ groups appear to reduce electrostatic repulsion (Figure 5c), thereby stabilizing the presence of four protonated nitrogen atoms in the solid state.

### Solid State NMR Analysis for T.Br-α

To complement X-ray diffraction analyses, the structure of **T.Br-α** was determined using NMR crystallography. Specifically, we determined the protonation state of the amine groups in the triptycene linker, and found that the sample comprises a mixture of charged and neutral amines. The ^1^H-^1^H DQ/SQ spectrum,^27^ shown in Figure 3, contains four resonances. The assignment is straightforward given four potential inequivalent protons, H1/H8, H3/6, and NH_3_ or NH_2_ groups. We assign the resonance at 10.8 ppm to an NH_3_ group, showing a single correlation to a resonance at 8.8 ppm. This resonance is assigned to H3/H6 due to the proximity to the NH_3_ group. Two additional correlations arise for the H3/H6 group at 6.9 and 6.1 ppm, which are pairwise assigned to the aliphatic H1/H8 and to the presence of a neutral amino group.

**Figure 3:**
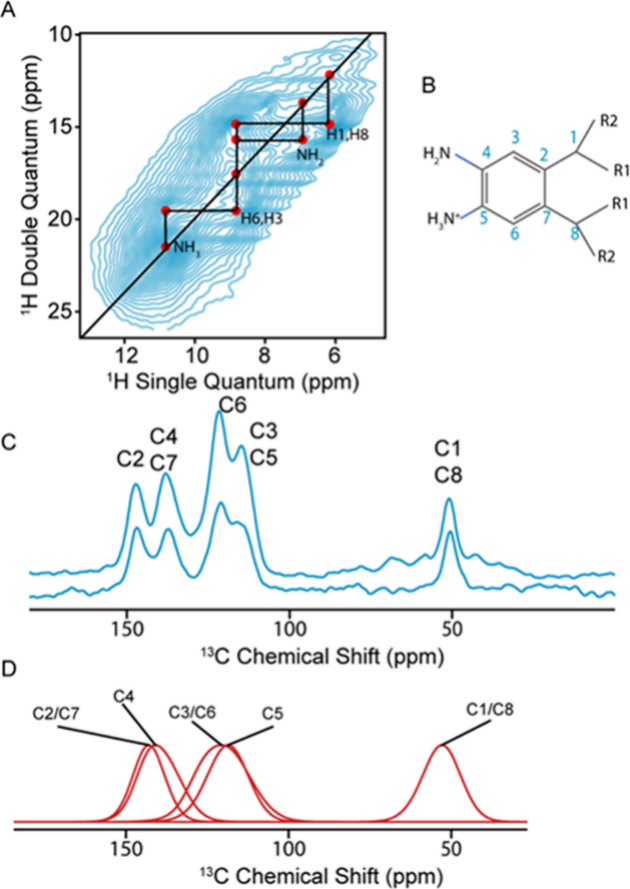
(a) Room temperature (RT) ^1^H-^1^H DQ/SQ spectrum of triptycene acquired at 21.1 T and 30 kHz MAS. Red dots indicate the correlation peaks observed for the corresponding assigned atomic sites. The diagonal black line represents the self-correlation peaks. The horizontal and vertical black lines indicate the correlations between the corresponding atomic sites. (b) Molecular fragment of triptycene representing the monomer unit. Blue numbers indicate the label of the atomic sites. R1 and R2 represent the other monomer units. (c) ^13^C CPMAS spectra acquired at 9.4T at 10 kHz and 8 kHz for the RT and 100K, respectively. The top spectrum is acquired at 100 K, and the lower is acquired at RT. D) The predicted distributions of possible chemical shifts for the different sites, using the database approach.^28^ The distributions are labeled with the corresponding atomic sites.

To verify the assignment, we exploited the large database of predicted shifts using machine learning models from Cordova et al.^28^ For each hydrogen atom, the possible distribution of chemical shifts is given for all the structures in the database with the same connectivity. From this database, the charged amine group has a predicted distribution centered at 9.6 ppm, which can be compared to the neutral amine, which has a predicted distribution centered at 5.9 ppm.

Likewise, the ^13^C resonances are sensitive to the protonation state of the amine groups. From the ^13^C CP MAS spectrum, we observe five shift distributions, which were again assigned using the database of predicted shifts. Notably, the predicted distributions for C5 and C4 are separated by 21 ppm, with C4 bonded to the neutral amino group, centered at 140 ppm, and C5 to the charged amino group, centered at 119 ppm. The quaternary aromatic carbons C2/C7 have a predicted distribution centered at 143 ppm, with the remaining aromatic carbons, C3/C6, at 120 ppm. The aliphatic C1/C8 are assigned to the distribution at 54 ppm, with the corresponding database distribution centered at 53.52 ppm. The experimental ^13^C and ^1^H spectra both correlate well with the database of predicted shifts, and we therefore confidently determine that the structure contains a mixture of NH_3_ and NH_2_.

To further validate the structure and to resolve ambiguous assignments, we compared against the calculated shifts from the single crystal X-ray structure of **T.Br-α**. We used the crystal structure to make models with various bromide and water positions such that the overall charge was zero. The computed shifts from these models allowed us to resolve the spectral assignment of the aromatics, where C2 was observed at 147.9 ppm and C7 at 137.9 ppm. We find that the resonance for C3 is at 115.1 ppm and for C6 at 121.8 ppm. Given the complete assignment of the carbons, we can then estimate the ratio of NH_3_ to NH_2_ to be approximately 2:1 at 100 K by using quantitative ^13^C direct polarization experiments with a recycle delay of 300 s. This 2:1 ratio is qualitative, given the spectral quality and structural complexity, but the charged amino groups occur more frequently. It is likely that the structure is significantly more complex than those modeled, due to the presence of water and bromide ions. From the DFT calculations, we note that the changes in water position or density can induce changes of up to 1 ppm for proton and up to 5 ppm for carbon chemical shifts. We postulate that the rearrangement of water molecules in the structure leads to the broad carbon-13 peaks observed which, at widths of roughly 500 Hz, are an order of magnitude larger than those observed for typical microcrystalline organic solids. This 2:1 ratio of NH_3_ to NH_2_ groups, neutralized by 4 bromide counterions, agrees with single crystal X-ray structure refinements for **T.Br-α** and its isostructural analog. We note that Xie et al. discuss full protonation of all six amines in their recent report on HAT.Cl,^26^ which does not agree with our combined NMR and X-ray analyses here.

### Crystal Structure Prediction

CSP was performed initially for **T.Br** assuming a fully protonated state for the cation combined with 6 Br- anions. The resulting landscape (Figure S20) displays no porous structures within the lowest 100 kJ mol^–1^. The results suggest that it is unlikely that a porous structure exists with all amines protonated, if this salt should prove synthesizable. Considering the experimental evidence for a partially protonated state for the triptycene linker, as discussed above, additional CSP calculations were performed to explore two further compositions with the 3+ and 4+ T (triptycene) cations, respectively, and the corresponding number of Br^–^ counter-anions. We found that porous structures emerge at lower energies (ca. 30 kJ mol^–1^ above the global energy minimum) with the 4+ protonation state and then increase again as the charge state is reduced to + 3, whereby the lowest energy porous structures appear at ca. 75 kJ mol^–1^ above the global energy minimum (Figure S20). The porous structures predicted on the 4+ CSP landscape are within the energy range where solvent stabilization could make these porous structures energetically competitive with the higher density global energy minimum. By contrast, the porous structures for the 6+ and 3+ cations fall outside of that range.

Thus, while these calculations, which include only ordered structures, cannot fully reproduce the observed disordered structure, the trend in energetics of porous structures supports the formation of a porous structure based on a 4+ T cation. More broadly, this lays the basis for qualitative computational screening of hypothetical salts for likely porosity in cases, such as here, where the experimental salt stoichiometry is not known *a priori*. The main disadvantage of this approach is the computational cost, which is already high for the three different protonation levels assessed here, and would double again for full assessment of all six hypothetical T.X cation possibilities ranging from + 1 to + 6. Realistically, it would require either substantial modifications to the CSP methodology or extremely large computational budgets to allow large-scale computational porosity screening of complex organic salts where the stoichiometry is not known.

### Gas Sorption Properties of T.Cl-α and T.Br-α

The voids in **T.Cl-α** make up 39.7% of the unit cell volume. A slightly smaller void volume was calculated for **T.Br-α** (37.9%). Both samples were activated at 70 °C under dynamic vacuum for 20 h. Xie et al. reported the use of supercritical CO_2_ to activate their Cl sample (HAT_Cl),^26^ but we found that this was unnecessary, possibly because our bulk material was made via a different synthesis route. **T.Cl-α** was stable to both heat and vacuum, but the bromide analogue, **T.Br-α**, exhibited even higher thermal stability. This was demonstrated by single crystal X-ray data of **T.Br-α**, which showed that no electron density was present within the voids after heating the crystals under dynamic vacuum at 70 °C for 20 h (see SI). Stability to activation is a widespread problem for porous organic salts,^4,5^ and for our first three N-MOFs, we were only able to achieve single crystal diffraction data for one compound (TAPT.Cl) in the form of a solvate.^6^ As such, the retention of single crystallinity in **T.Br-α** after activation is noteworthy.

**T.Cl-α** showed higher mass-normalized gas uptakes than **T.Br-α** for all gases studied (CO_2_, N_2_, H_2_), probably because of the higher atomic weight of bromine coupled with the slightly larger void volume calculated for **T.Cl-α**. The maximum CO_2_ uptake for **T.Cl-α** was 4.5 mmol g^–1^ at 273 K (Figure S10), while the maximum N_2_ uptake was 5.7 mmol g^–1^ (Brunauer–Emmett–Teller surface area (SA_BET_) = 466 m^2^ g^–1^; Figure S9), again with a Type I isotherm.

Figure 4 shows the gas sorption isotherms for **T.Br-α**, the more stable of these triptycene salts, showing that the gas sorption for all three gases greatly exceeds that reported for our first three isoreticular N-MOFs.^6^

**Figure 4:**
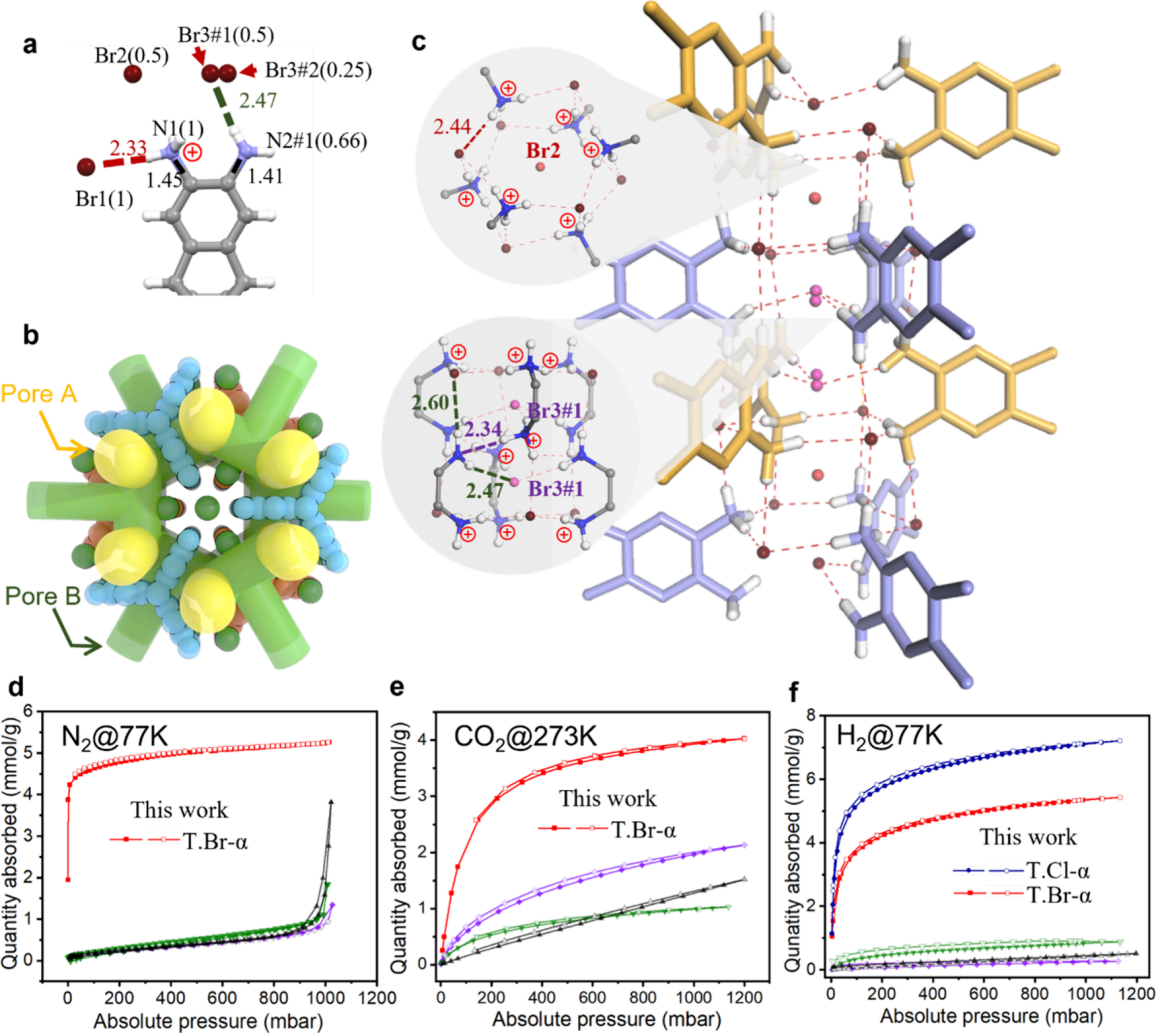
Single-crystal X-ray structure and sorption isotherms for **T.Br-α**. a) Position and occupancy disorder of bromide anion and charge-assisted H -bonds with NH_3_^+^ or NH_2_ group in triptycene. b) Space-filling structural model for **T.Br-α** showing the interconnected 3-D pore channel with two distinct pores (Pore A: yellow, Pore B: green). c) The H-bonding chains in single-crystal structures of **T.Br-α** with two distinct hydrogen-bonding patterns: H-bonding cluster formed by Br1(1) and NH_3_^+^ groups (red dash line) by containing Br^–^(0.5) inside cavity; H-bonds between NH_2_ and NH_3_^+^ groups (purple dash line), and H-bonds between NH_2_ and Br3#1 (green dash line). Sorption isotherm comparisons for **T.Br-α** (red) with our first generation of N-MOFs;^6^ TAPT.Cl (purple), TTBT.Cl (black) and TT.Br (green). d) N_2_ @77 K_,_ e) CO_2_@273 K and f) H_2_@77 K for **T.Cl-α** (blue) and **T.Br-α** (red). Filled symbols are adsorption isotherms, open symbols are desorption isotherms.

The H_2_ sorption in these materials is particularly remarkable (Figure 4f). **T.Cl-α** shows a H_2_ uptake of 7.2 mmol g^–1^ at 77 K and 1 bar. This compares favorably with many MOFs under these conditions.^29–31^ A few MOFs have been reported with higher H_2_ uptakes—for example, **T.Cl-α** exhibits just half the H_2_ uptake of UPC-501,^32^ a 2-fold interpenetrated zinc MOF (14.8 mmol g^–1^ at 77 K and 1 bar)—but UPC-501 was specifically designed to have the synergistic effects of pore size (small windows) combined with high surface area (2394 cm^3^ g^–1^, which is around 6 times higher than **T.Cl-α**) to promote hydrogen sorption. The H_2_ sorption in **T.Cl-α** is anomalously high for a material that does not combine optimal pore sizes (6–7 A≈) and high surface area (>2000 cm^3^ g^–1^).^33^ For comparison, a neutral porous molecular crystal reported by Kadhum et al.^34^ showed a H_2_ uptake of 3.9 mmol g^–1^ at 10 bar and 77 K and Comotti et al.^35^ reported a dipeptide porous crystal with a H_2_ uptake of 2.1 mmol g^–1^ at 77 K and 10 bar.^34,35^ As such, **T.Cl-α** shows substantially higher H_2_ uptakes than these neutral molecular crystals at 10% of the gas pressure. Further comparisons of the H_2_ storage in **T.Cl-α** with other porous materials can be found in the SI, Table 2.

### Gas Sorption Properties of T.Cl-β

We next investigated the gas sorption properties of **T.Cl-β** using CO_2_, N_2_, and H_2_ as sorbate molecules after removing the guests from the pores at 70 °C under dynamic vacuum for 20 h. PXRD measurements (Figure S8) suggested that the solvate structure shown in Figure 5 was retained. **T.Cl-β** showed a maximum CO_2_ uptake of 4.0 mmol g^–1^ at 273 K, making it also one of the most porous organic salts reported so far. It absorbs twice as much CO_2_ than TAPT.Cl, which was the most porous of our first three N-MOFs under these conditions (Figure 5e)^.6^ **T.Cl-β** also adsorbed N_2_ at 77 K, showing a Type 1 isotherm (Figure 5d); again, our first-generation N-MOFs were all nonporous to N_2_ at this temperature^.6^ **T.Cl-β** showed an N_2_ uptake of 4.3 mmol g^–1^ at saturation (SA_BET_ = 413 m^2^ g^–1^). Nitrogen adsorption in porous salts is rare; indeed, in the three decades since Ward and co-workers pioneered porous salts,^36–38^ for the systems that have been shown to have permanent porosity this is in most cases limited to CO_2_, with them showing no N_2_ uptake.^18,22,23^ Only a few systems have showed porosity to N_2_, the most notable examples being TPMA-Cl/MTBPS (5.6 mmol g^–1^)^39^ and *p*-G_2_BDS (5 mmol g^–1^).^40^

**Figure 5:**
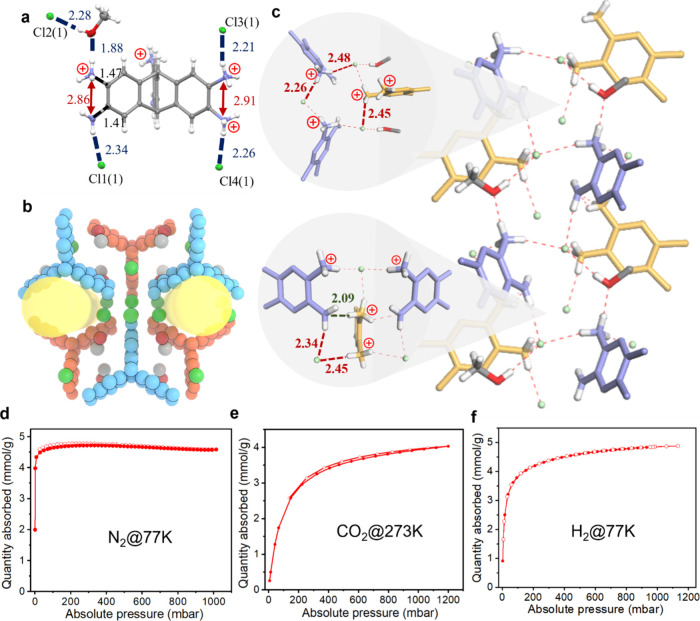
Single-crystal X-ray structure (MeOH solvate) and sorption isotherms (activated material) of a porous polymorph, **T.Cl-β**, highlighting (a) the difference in the bond lengths of H_3_^+^N – Cl and H_2_N – Cl, the difference in distance between two NH_3_^+^ groups and (NH_2_ and NH_3_^+^) pairs, the H bonds between Cl^–^ and NH_3_^+^, Cl^–^ and NH_2_, and methanol and Cl^–^; b) crystal packing of **T.Cl-β** showing 1-D pores along the *a* axis; c) The H-bonding chains in **T.Cl-β** with two distinct hydrogen-bonding patterns: H-bonding cluster (top) formed by Cl and NH_3_^+^ groups (red dash line); H-bonds (bottom) between NH_2_ and NH_3_^+^ groups (green dash line), and H-bonds between NH_2_ and Cl (red dash line). The formation of hydrogen bonds between NH_2_ and NH_3_^+^ mitigates the electrostatic repulsion between adjacent NH_3_^+^ groups. Sorption isotherm comparisons for **T.Cl-β** (red): d) N_2_@77 K, e) CO_2_@273 K, and f) H_2_@77 K. Filled symbols are adsorption isotherms, open symbols are desorption isotherms.

**T.Cl-β** also showed a high H_2_ uptake of 4.9 mmol g^–1^ (10 g/L) at 77 K and 1 bar (Figure 5f).

### Origin of H_2_ Capacity in Porous Triptycene N-MOFs

We hypothesized that the high H_2_ uptakes in **T.Br-α**, **T.Cl-α**, and **T.Cl-β** are due to the polar pore channels, which are all lined with halide anions (Figure 6a,b). By contrast, our previous N-MOF, **TTBT.Cl**, has two types of pores (A and B); the A pores are ionic, but in the larger B pores, which make up most of the pore volume, the halide anions are much less exposed.

**Figure 6:**
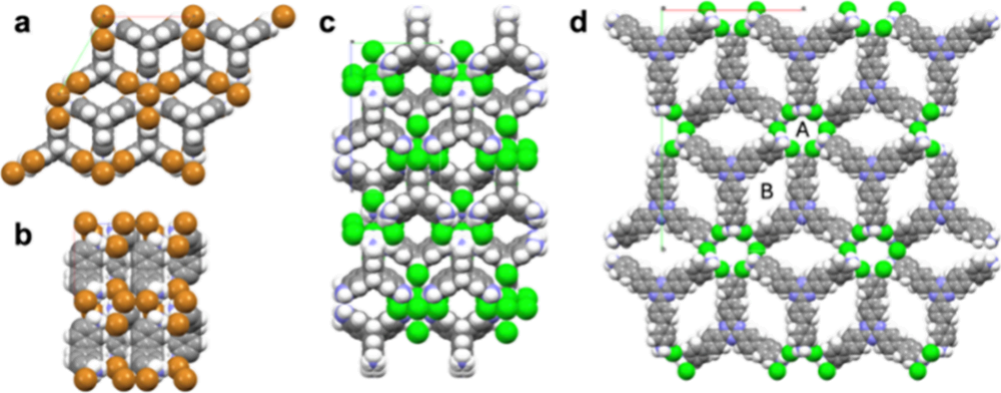
3-D porosity in **T.Br-α** shown along the crystallographic *a*-axis (a) and *b*-axis (b) with halide disorder removed, and 1-D porosity in **T.Cl-β** shown along the crystallographic *a*-axis (c). In both materials, all of the pore channels are polar and lined with halide ions. By contrast, our previous N-MOF, **TTBT.Cl**, has two types of pores (d) that are not interconnected; a polar, halide-lined pore (A) and a less polar pore (B), which constitutes most of the total pore volume.

To probe the superior performance of **T.Cl-α** and **T.Br-α**, for H_2_ sorption, which share the same general crystal structure (Figure 6a–b), over **TTBT.Cl** (Figure 6d), we used Monte Carlo sampling accelerated by the use of machine learned interatomic potentials, combined with DFT geometry optimizations (FHI-aims,^41^ PBE^42^ + D3­(BJ) dispersion correction^43^) to locate and refine adsorption sites for H_2_ and CO_2_ in these three porous hosts. For H_2_, **T.Cl-α** and **T.Br-α** each exhibit a single, strongly preferred binding site (Figures S14–S15), whereas **TTBT.Cl** presents four distinct preferred sites (Figure S16). Binding-energy calculations show that the mean stabilization per H_2_ follows the order **T.Cl-α** > **T.Br-α** > **TTBT.Cl** (Table 1). This ordering is identical whether we consider the energy gain from adsorption of an isolated H_2_ molecule or the average stabilization across the sampled H_2_ population, and it is consistent with the H_2_ uptake trend reported in Figure 4f; that is, we can rationalize the observed experimental order of H_2_ uptakes. CO_2_ binding sites were identified, too (Figures S17–S19). Although steric constraints produce different CO_2_ geometries in **T.Cl-α** and **T.Br-α**, the calculated binding energies again support the experimental trend shown in Figure 4e (Table 1). These data are in line with previous reports that H_2_ storage capacities in MOFs are generally proportional to adsorption energies for low loadings.^44^

**Table 1:** Calculated Preferred Binding-Site Adsorption Energies (ML-Accelerated Monte Carlo Sampling; DFT Optimizations: FHI-aims, PBE+D3­(BJ))^a^

host	H_2_ BE^b^ (kJ·mol^–1^)	avg. H_2_ BE (kJ·mol^–1^)	CO_2_ BE (kJ·mol^–1^)
T.Cl-α	11.08	10.56	29.84
T.Br-α	8.98	9.65	30.30
TTBT.Cl	7.25^c^	8.42	22.99

a Full energy lists and details are provided in the ESI.

b **BE**: binding energy,

c Averaged over four highest binding sites.

Calculated Hirshfeld partial charges on adsorbed H_2_ molecules were < 0.02 e in all cases, consistent with a dispersion-dominated physisorption picture reported previously for H_2_ adsorption in MOFs;^45^ weak polarization thus implies only a minor role for classical electrostatics, even though these materials are organic salts, and this helps to rationalize the enhanced N_2_ uptake in Figure 4d. Complete numerical data, site geometries, and computational details are provided in the ESI. Similar to MOFs of comparable pore diameter, the long-range interaction potentials of the pores in **T.Cl-α** and **T.Br-α** act constructively to yield strong H_2_ binding.^45^ It is also noteworthy that the pore sizes of **T.Cl-α** and **T.Br-α** (∼0.5 nm) are close to those of well-characterized nanoporous materials (∼0.6 nm) that were studied in a similar context.^46^

## Conclusions

We report three new triptycene porous halide salts, **T.Br-α**, **T.Cl-α**, and **T.Cl-β**, with gas sorption properties that surpass previous porous organic salts. We also show the first example of porous N-MOF polymorphs (**T.Cl-α**, and **T.Cl-β**). The high stability of these materials to activation was demonstrated through gas sorption studies. Notably, they show H_2_ uptakes (1 bar, 77 K) that exceed other porous molecular crystals as well as many bonded frameworks. DFT binding-energy analysis indicates that the superior H_2_ and N_2_ adsorption of the new N-MOFs is driven primarily by London-dispersion interactions and geometric confinement within subnanometre pores, with classical electrostatic contributions playing only a minor role, even though these materials are salts. This is a further illustration that N-MOFs can offer useful and distinctive physical properties, and that there is broad scope for fine-tuning this, both by changing crystal packing, as in the **T.Cl** polymorphs, and by changing composition, for example by varying anions.

## Supplementary Material





## Data Availability

Computational data related to the crystal structure prediction calculations, H_2_ and CO_2_ adsorption calculations is available at: https://doi.org/10.5258/SOTON/D3692.

## Notes

**Supporting Information**, including methods, further NMR details and characterization of the materials (PDF)
Further details of NMR studies and the associated coordination files (ZIP)

## ABBREVIATIONS

N-MOF: non-metal organic frameworks
CPOS: crystalline porous organic salts
MOF: metal organic framework
CSP: crystal structure prediction
