# Supplementary material for: Polar Triptycene-Based Nonmetal Organic Frameworks Show Enhanced Hydrogen Adsorption
Source: J Am Chem Soc. 2025 Oct 16;147(43):39351–8. doi: 10.1021/jacs.5c11317 (PMC12576833; doi:10.1021/jacs.5c11317)
Supplement: Supplementary file 1 [file ja5c11317_si_001.pdf]

Polar triptycene-based non-metal organic frameworks show enhanced hydrogen adsorption

Megan O'Shaughnessy,[1] Hang Qu,[1] Xue Wang,[1] Jacob B. Holmes,[2] Lyndon Emsley,[2] Joseph Glover,[3] Roohollah Hafizi,[3] Graeme M. Day,[3] and Andrew I. Cooper[1*]

[1] Department of Chemistry, University of Liverpool, Liverpool, L69 7ZD, United Kingdom

[2] Institut des Sciences et Ingénierie Chimiques, École Polytechnique Fédérale de Lausanne (EPFL), CH-1015 Lausanne, Switzerland; National Centre for Computational Design and Discovery of Novel Materials MARVEL, École Polytechnique Fédérale de Lausanne (EPFL), CH-1015 Lausanne, Switzerland.

[3] School of Chemistry and Chemical Engineering, University of Southampton, Southampton, SO17 1BJ, United Kingdom

General Information

Materials: All reagents were obtained from Sigma-Aldrich, Manchester Organics, Fluorochem and Alfa Aesar and used as received. All gases for sorption analysis were supplied by BOC at a purity of ≥ 99.9%

NMR: ^{1}H NMR spectra were recorded at 400 MHz on a Bruker Advance 400 NMR spectrometer. Chemical shifts are reported in ppm with reference to internal residual protonated species of the deuterated solvents used for ^{1}H analysis.

Solid state NMR: The ^{13}C CP MAS spectra were acquired on a commercial Bruker Avance III spectrometer operating at 9.4T (101MHz 13C Larmor frequency) using a 3.2mm H/X/Y LTMAS probe at 10kHz MAS for the room temperature and 8 kHz for the 100K spectra. The quantitative ^{13}C direct polarization experiments with a recycle delay of 300 seconds on a commercial Bruker Avance III spectrometer operating at 11.7T (126 MHz 13C Larmor frequency) at 10 kHz MAS at 100K. The 1H-1H DQ/SQ Bruker Avance Neo spectrometer operating at 21.1 T (900 MHz 1H Larmor frequency) using 1.3mm H/C/N/D DVT probe at 30

kHz MAS at room temperature. All spectra were externally referenced to adamantane CH2 group at 1.87 ppm for ^{1}H and adamantane CH group at 38.48 ppm for ^{13}C.

DFT Calculations: The atomic positions and unit cell parameters obtained from X-ray diffraction were used as starting points for DFT chemical shift calculations. Due to ambiguity in the bromide and water positions in the structure, various models with different bromide positions and permutations of the water positions were built before geometry optimization. Structures were generated with 1 of 5 potential inequivalent bromide positions as observed in the crystal structure. The water positions were taken as permutations of the inequivalent water molecules observed in the crystal structure resulting in 1-3 inequivalent waters within each starting structure before geometry optimization. All structures that reached convergence during geometry optimization were used for NMR chemical shift calculations, and coordinate files are given in the supporting materials. The number of bromide ions used equalled the number of NH_3 to maintain an overall charge of zero. The geometry of all atoms was then optimized with fixed cell parameters using the plane-wave DFT software Quantum ESPRESSO.[1,2] The optimizations were performed at the PBE[3,4] level of theory using the Grimme D3 dispersion correction[5,6] and projector augmented wave pseudopotentials, C.pbe-n-kjpaw_psl.1.0.0.UPF, H.pbe-kjpaw_psl.1.0.0.UPF, Br.pbe-n-kjpaw_psl.1.0.0.UPF, N.pbe-n-kjpaw_psl.1.0.0.UPF, O.pbe-nl-kjpaw_psl.1.0.0.UPF. The wavefunction and charge density energy cutoffs were set to 80 Ry and 640 Ry, respectively. A 4x4x5 Monkhorst−Pack grid of k-points corresponding to a maximum spacing of 0.13 $Å^{-1}$ was used. The GIPAW NMR[7] calculations were performed using the same parameters as the geometry optimization. The calculated shieldings were rescaled using an on-the-fly regression using the average shift from each relaxed structure.

Gas sorption analysis: Samples (above 100 mg) of **T.Br-α**, **T.Cl-α** and **T.Cl-β** were activated for sorption measurements at 70 °C under reduced pressure for 20 hours. Nitrogen and hydrogen isotherms were collected at 77 K using an ASAP2420 volumetric adsorption analyser (micrometrics instruments Corporation). Carbon dioxide isotherms were collected up to a pressure of 1200 mbar on a micrometrics ASAP2020 volumetric adsorption analyser at 273 K. Carbon dioxide isotherm at 195 K were collected using Micromeritics 3flex volumetric adsorption analyser. See **Methods**, main text, for further details.

Powder X-ray Diffraction (PXRD): Powder X-ray diffraction data were collected in transition mode on powder samples held on thin Mylar film in aluminium well plates on a Panalytical Empyrean diffractometer equipped with a high throughput screening XYZ stage, X-ray focusing mirror, and PIXcel detector, using Cu-Kα (λ = 1.541 Å) radiation.

Single crystal X-ray Diffraction: SC-XRD data sets were collected on a Rigaku MicroMax-007 HF rotating anode diffractometer (MoKα radiation, λ = 0.71073 Å) or XtaLAB Synergy (Dualflex, HyPix, CuKα radiation, λ = 1.54184 Å). The raw data were reduced by CrysAlisPro 1.171.43.90. The structures were solved by the SHELXT with Intrinsic Phasing and refined on F_2 by full-matrix least-squares methods with the SHELXL and OLEX2 was used as GUI. The detailed crystal parameters are listed in the Supplementary Table S4 and the supporting CIFs.Refinement details: all non-hydrogen atoms were refined anisotropically. Hydrogen atoms were placed at calculated positions using the riding model and refined isotropically. The instructions AFIX 43, 93 and 137 were used for the hydrogen atoms on the aromatic C-H, NH_2 group and NH_3^+ group, respectively, with the parameter of Uiso = 1.2 Ueq. For all crtystal structure, a satisfactory disorder model for the solvent molecules was not found, therefore the PLATON/SQUEEZE routine was used to mask out the disordered density. For the crystal structure of T.Br, its chemical composition was determined based on solid-state NMR spectra. Consequently, the N2 atom was split into two sites with occupancies of 2/3 (N2#1) and 1/3 (N2#2). In addition, the EADP, EXYZ, RIGU and SIMU constrains were applied to N2#1 and N2#2. Due to positional disorder, the occupancies of Br2, Br3#1, and Br3#2 were determined to be 0.5, 0.5, and 0.25, respectively. The similar operations were also applied to T.Cl.

Experimental section

Synthesis and characterisations of salts

2,3,6,7,14,15-Hexaaminotriptycene.4Br: HBr solution (48.wt% in water, 0.15 mL) was added to a THF solution (5.0 mL) of N2 ,N3 ,N6 ,N7 ,N14,N15-Hexakis(diphenylmethylene)triptycene (100 mg, 0.08 mmol) and the mixture was stirred at room temperature for 30 minutes. The precipitate was isolated by filtration, washed twice with THF, (40 mL) ethyl acetate, and hexane (20 mL) sequentially, and dried under vacuum to give the hexaammoniumtriptycene bromide salt 3 as an off-white solid (39 mg, 91%). ^{1}H NMR (400 MHz, CD_3OD), δ 7.90 (protonated amine), 7.14 (s, 6H), 5.30 (s, 2H) ppm (Figure S1).

2,3,6,7,14,15-Hexaaminotriptycene.4Cl was synthesised using a previously reported method.[8] ^{1}H NMR shown in Figure S2.

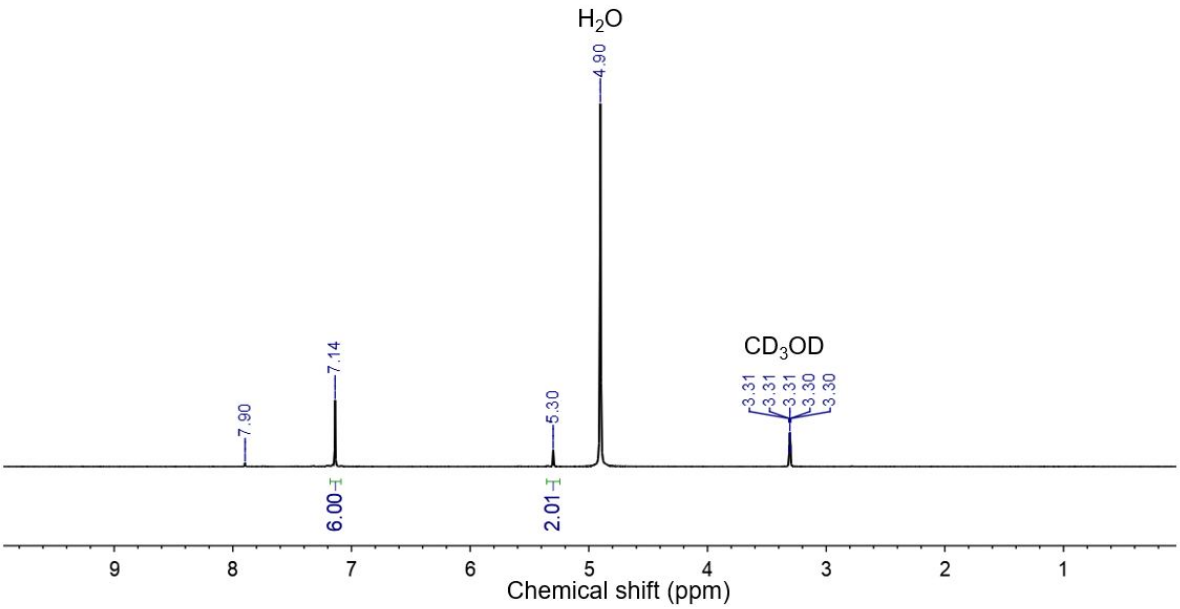

Figure S1. ^{1}H NMR for 2,3,6,7,14,15-Hexaaminotriptycene.4Br in CD$_3$0D.

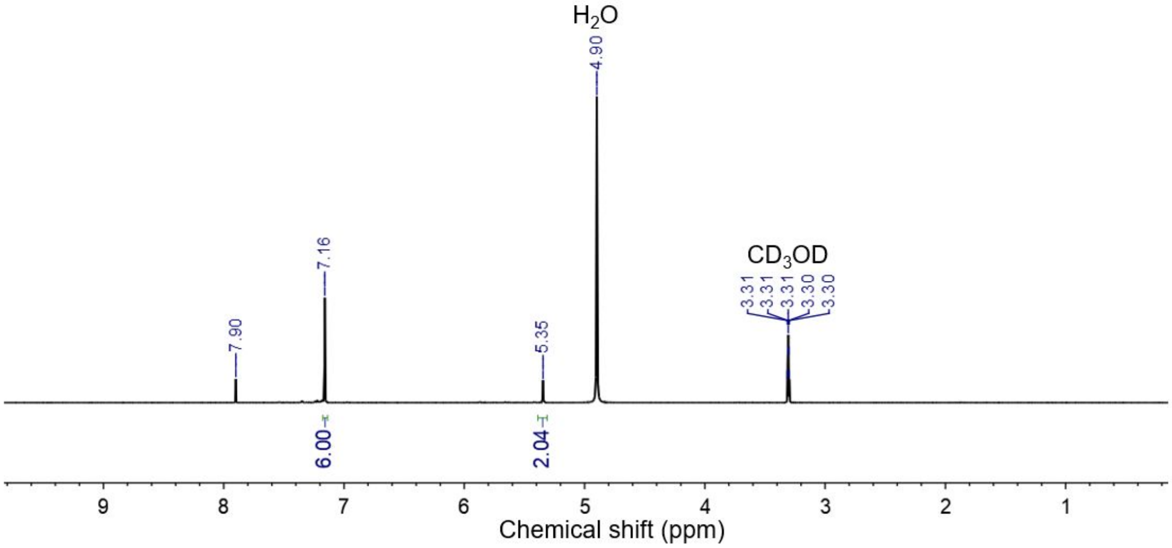

Figure S2. ^{1}H NMR for 2,3,6,7,14,15-Hexaaminotriptycene.4Cl in CD$_3$0D

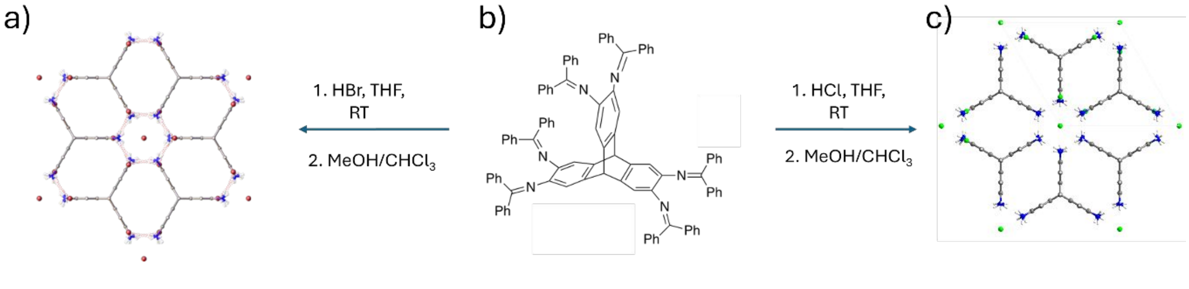

Scheme 1. Reaction scheme to make the **T.Br-α** (a) shown as the crystal packing structure and **T.Cl-α** (c) shown as the crystal packing structure, directly from the intermediate (b) N2 ,N3 ,N6 ,N7 ,N14,N15-Hexakis(diphenylmethylene)triptycene-2,3,6,7,14,15-hexaamine without needing to make the unstable 2,3,6,7,14,15-Hexaaminotriptycene.

T.Br-α; Single crystals of **T.Br-α** were grown through dissolving a powder sample of T.Br in MeOH (5 mg/ 0.2 mL) and placing within a larger vial containing Chloroform and leaving it sealed for ~ 16 hours. Single crystals of T.Br-α were activated on a gas sorption instrument at 70 ^{0}C for 18 hours and were then rapidly (within 1 -2 minutes) placed into oil for collection on a Rigaku MicroMax-007 HF rotating anode diffractometer .The single crystal data showed that the crystal was fully activated through a lack of electron density within the channels. (Figure S3).

T.Cl-α;Single crystals of T.Br-α were grown through dissolving a powder sample of T.Br in MeOH (5 mg/ 0.2 mL) and placing within a larger vial containing Chloroform and leaving it sealed for ~ 16 hours.

T.Cl-β; Single crystals of **T.Cl-β** were grown by dissolving T.Cl in Methanol (5 mg/ 0.6 mL) and placing within a larger vial containing THF, which was left sealed for 2 days resulting. Alternatively, single crystals of **T.Cl-β** were grown by dissolving a powder sample of T.Cl in Methanol (5 mg/0.3 mL). The solution was then added to 0.1 mL of benzonitrile in a 3 mL vial. The MeOH was left to evaporate off, leaving crystals of **T.Cl-β** by the next day.

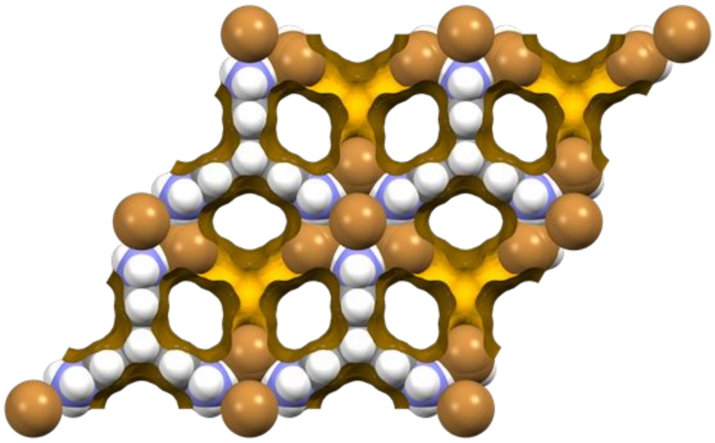

Figure S3. Crystal packing of **T.Br-α** down the *c* axis. Surface contact voids are shown in yellow using a probe radius of 1.2 Å and grid spacing of 0.1 Å. The voids make up 39.2 % of the unit cell volume and gives a surface area 644.97 Å^3.

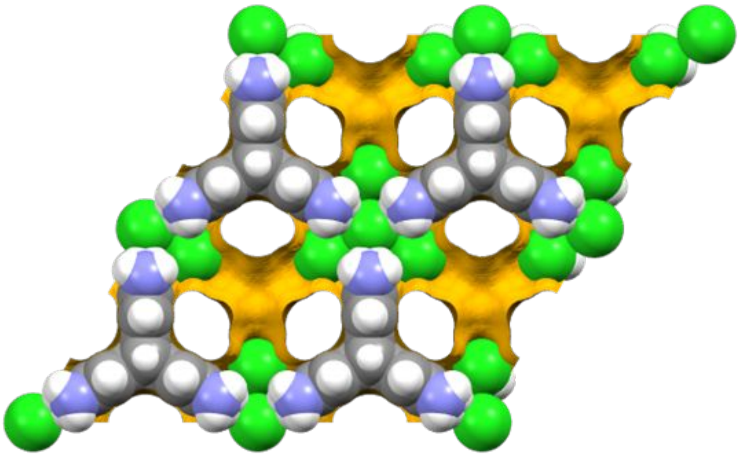

Figure S4. Crystal packing of **T.Br-α** down the *c* axis. Surface contact voids are shown in yellow using a probe radius of 1.2 Å and grid spacing of 0.1 Å. The voids make up 39.2 % of the unit cell volume and gives a surface area 644.97 Å^3.

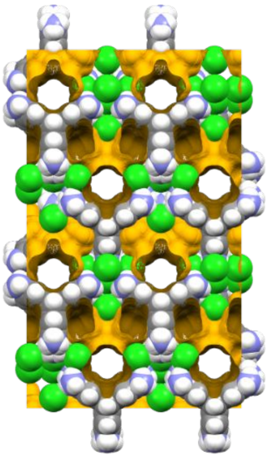

Figure S5. Crystal packing of **T.Cl-β** down a axis. Surface contact voids are shown in yellow using a probe radius of 1.2 Å and grid spacing of 0.1 Å. The voids make up 35.9 % of the unit cell volume and gives a surface area 1133.55 Å^3.

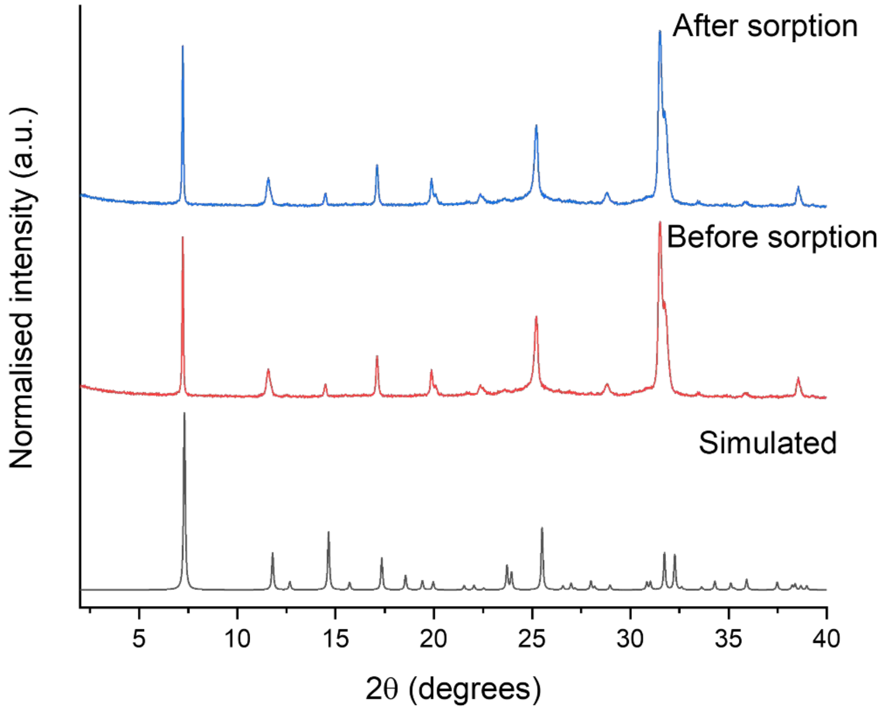

Figure S6. Simulated PXRD pattern for **T.Br-α** (black), b) PXRD pattern for experimental sample before isotherms (red), c) PXRD pattern after sorption measurements.

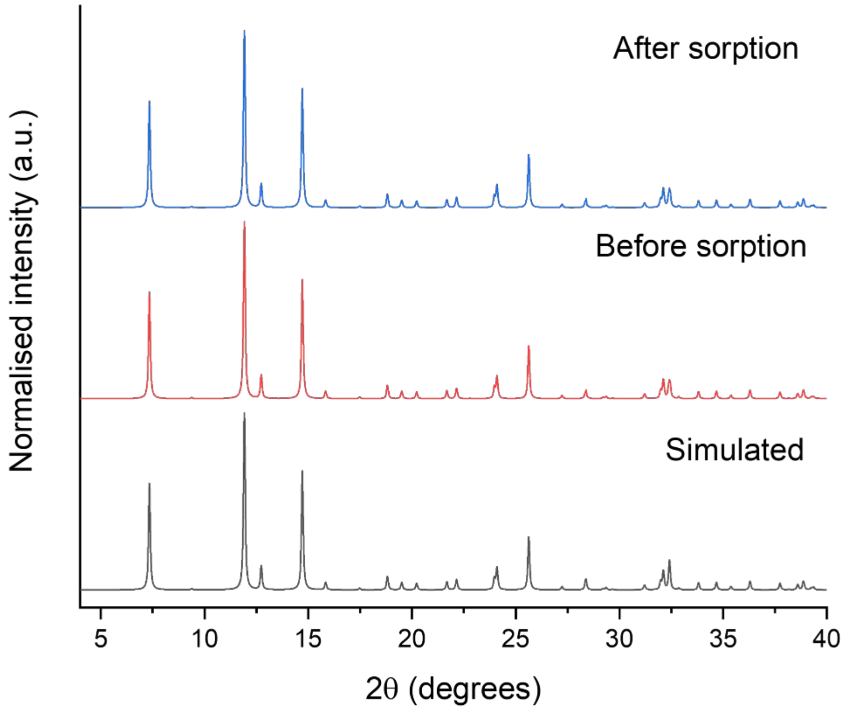

Figure S7. Simulated PXRD pattern for **TCI-α** (black), b) PXRD pattern for experimental sample before isotherms (red), c) PXRD pattern after sorption measurements.

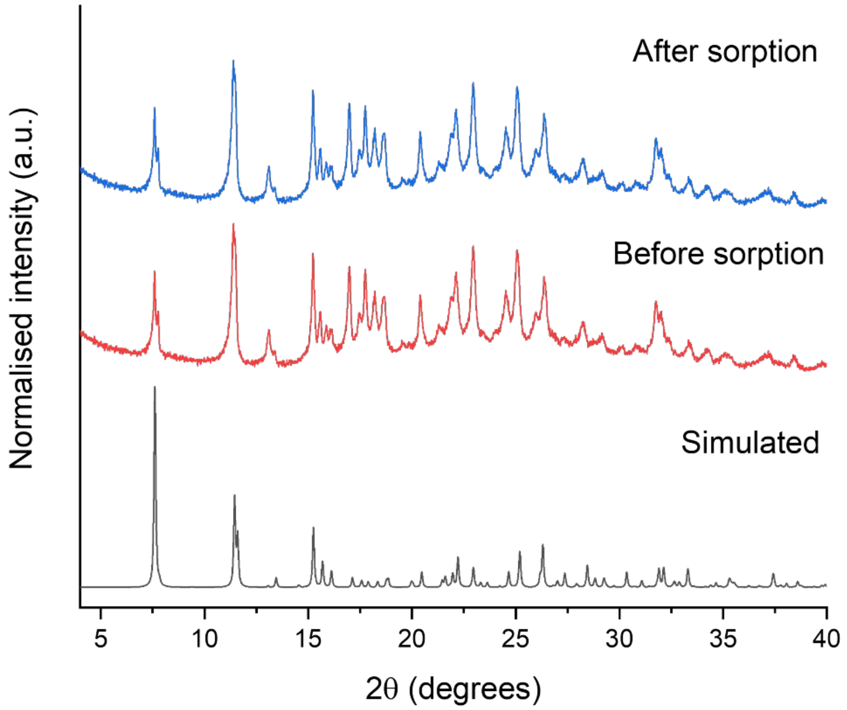

Figure S8. Simulated PXRD pattern for **T.Cl-β** (black), b) PXRD pattern for experimental sample before isotherms (red), c) PXRD pattern after sorption measurements.

Table 1. Crystallography tables for crystals

Molecule	**T.Br-α**	**T.Cl-α**	**T.Cl-β**
λ [Å]	1.54184	1.54184	0.71073
Collection Temperature	100 K	100 K	104 K
Formula	$C_{20}H_{24}Br_4N_6$	$C_{20}H_{24}Cl_4N_6$	$C_{22}H_{32}Cl_4N_6O_2$
Mr [g mol-1]	668.09	490.25	554.33
Crystal Size [mm]	0.16 x 0.093 x 0.056	0.1 x 0.1 x 0.1	0.19 x 0.18 x 0.16
Crystal System	Trigonal	Trigonal	Orthorhombic
Space Group	P -3 m 1	P -3 m 1	P n m a
a [Å]	13.9561(2)	13.8927(3)	10.3453(3)
b [Å]	13.9561(2)	13.8927(3)	13.5403(4)
c [Å]	9.5544(2)	9.4254(3)	22.5759(7)
A [°]	90	90	90
β [°]	90	90	90
γ [°]	120	120	90
V [Å^3]	1611.62(6)	1575.45(8)	3162.40(16)
Z	2	2	4
D_{calcd} [g cm^{-3}]	1.377	1.033	1.164
μ [mm^{-1}]	6.211	3.531	0.401
F(000)	652.0	508.0	1160
2θ range [°]	7.314 to 149.334	7.348 to 133.094	3.508 to 61.504
Reflections collected	18528	24515	17430
Independent reflections, R_{int}	1245, 0.0347	1058, 0.0717	4218, 0.0611
Obs. Data [$I > 2\sigma(I)$]	1173	1033	3630
Data /restraints / parameters	1245 / 24 / 73	1058 / 24 / 71	4218 / 0 / 177
Final R1 values ($I > 2\sigma(I)$)	0.0492	0.0686	0.0405
Final R1 values (all data)	0.0510	0.0701	0.0459
Final Wr(F_2) values (all data)	0.1385	0.1837	0.1154
Goodness-of-fit on F^2	1.070	1.114	1.053
Largest difference peak and hole [e.A^{-3}]	1.19/-0.76	0.60/-0.45	0.60/-0.59
CCDC	2466226	2466227	2466228

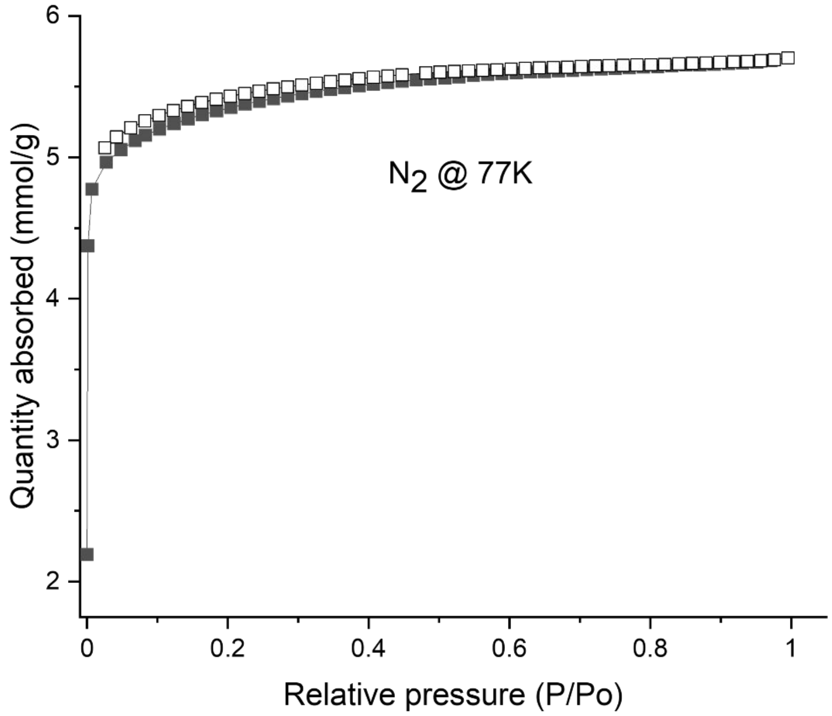

Figure S9. N_2 isotherm for **T.Cl-α** at 77 K, filled symbols are for absorption and empty symbols are for desorption.

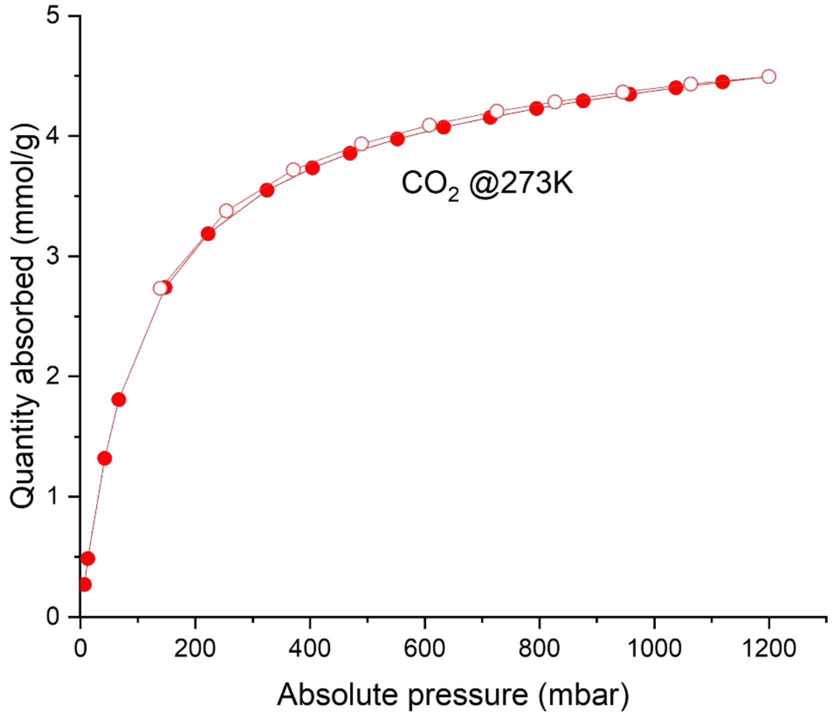

Figure S10. CO_2 isotherm for **T.Cl-α** at 273 K, filled symbols are for absorption and empty symbols are for desorption.

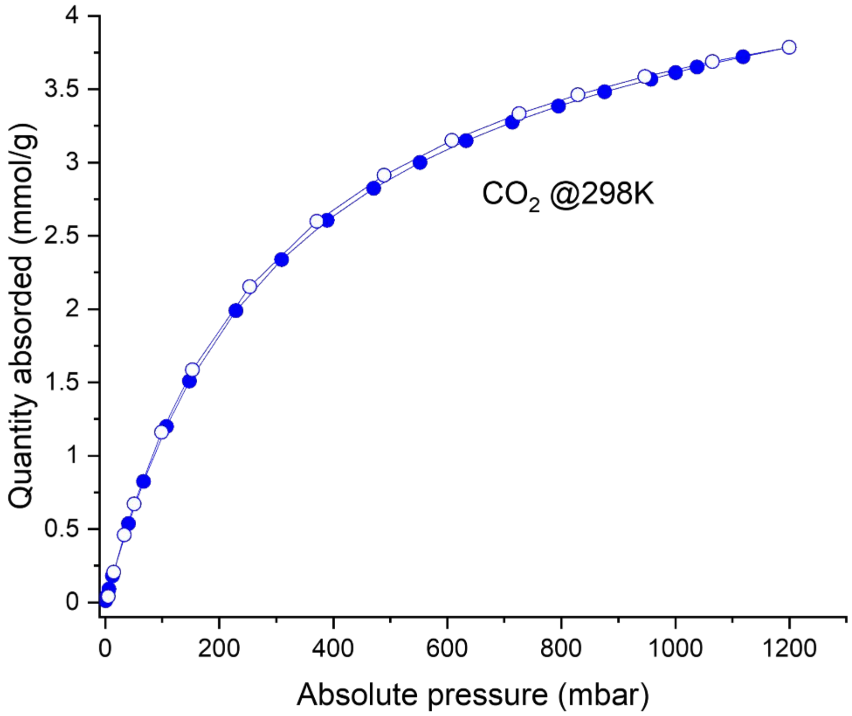

Figure S11. CO_2 isotherm for **T.Cl-α** at 298 K, filled symbols are for absorption and empty symbols are for desorption.

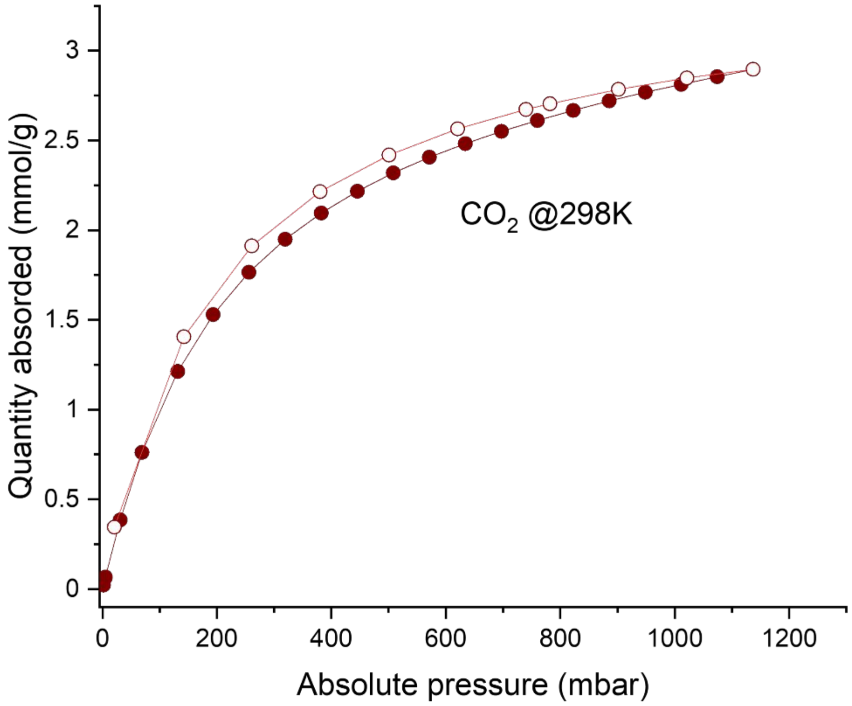

Figure S12. CO_2 isotherm for **T.Br-α** at 298 K, filled symbols are for absorption and empty symbols are for desorption.

Table 2. Comparisons of hydrogen storage properties of different materials under varying conditions.

Materials	S_{BET} (m^2 g^{-1})	Pore volume (cm^3 g^{-1})	Conditions	H_2 uptake (mmol/g)	H_2 uptake (g/L)	Reference
T.Cl-α	466	0.198	77K/1bar	7.2	14.5	*This work*
**HOFs**						
RP-H100	2,383	1.11	77K/100bar		54.8	Zhang, R., Daglar, H., Tang, C. *et al. Nat. Chem.* **16**, 1982–1988 (2024)
RP-H101	3,526	1.35	77K/100bar		56.5	Zhang, R., Daglar, H., Tang, C. *et al. Nat. Chem.* **16**, 1982–1988 (2024)
[(H_2dobpdc)(GuaH)$_2$]	N/A	N/A	77K/1bar	0.4		Song, J. H.; Jeon, B. H.; Kang, D. W. *CrystEngComm* **2024**, *26* (18), 2342-2345
ZJU-HOF-5a	N/A	N/A	77K/100bar		43.6	J.-X. Wang, X. Zhang, C. Jiang, T.-F. Zhang, J. Pei, W. Zhou, T. Yildirim, B. Chen, G. Qian, B. Li, *Angew. Chem.*

						Int. Ed. 2024, 63, e202411753.
**MOFs**						
UPC-501	2394	0.935	77K/1bar	14.8		Li, F.-G.; Liu, C.; Yuan, et al. *CCS Chemistry* **2021**, *4* (3), 832-837.
MOF-5	3362	N/A	77K/1bar	6.60		Rowsell, J. L. C.; Yaghi, O. M. *J. Am. Chem. Soc.* **2006**, *128*, 1304–1315
$[Ni_3(pzdc)_2(ade)_2]$	106	N/A	77K/1bar	~2.25		Chiu, N. C.; Compton, D.; Gładysiak, A.; et al. *ACS Applied Materials & Interfaces* **2023**, *15* (45), 52788-52794.
Uio-67	2360	0.91	77K/100bar	31.14		Villajos, J. A. Carbon Res. (MDPI) **2022**, **8**, 5.
IRMOF-9	1904	0.9		5.58		Rowsell, J. L. C.; Yaghi, O. M. *J. Am. Chem. Soc.* **2006**, *128*, 1304–1315
ZIF-8	1630	0.64	77K/1bar	6.35		Park, K. S.; Ni, Z.; Côté, A. P. et al. *Proceedings of the National Academy of Sciences* **2006**, *103* (27), 10186-10191.
MOF-177	4526	N/A	77K/1bar	6.25		Rowsell, J. L. C.; Millward, A. R.; Park, K. S.; Yaghi, O. M. *Journal of the American Chemical Society* **2004**, *126* (18), 5666-5667.
Ni50Co-IRMOF-74	1913	N/A	298K/100bar		3.1	H. Montes-Andrés, G. Orcajo, C. Martos, J. A. Botas, G. Calleja, *Int. J. Hydrogen Energy* **2019**, 44, 18205–18213
**COFs**						
COF-5	1670	N/A	77K/35bar	17.8		Furukawa, H.; Yaghi, O. M. J. Am. Chem. Soc. **2009**, 131, 8875–8883.
COF-102	3472	1.22	77K/35bar	35.9	40.4	Han, S. S.; Furukawa, H.; Yaghi, O. M.; Goddard, W. A. J. Am. Chem. Soc. **2008**, 130,
COF-103	4210	1.36	77K/35bar	35	49.8	Furukawa, H.; Yaghi, O. M. J. Am. Chem. Soc. **2009**, 131, 8875–8883.

Computational Methods

To rationalize the enhanced H_2 uptake observed in **T.Br-α** and **T.Cl-α** as compared to our previously studied N-MOF **TTBT.Cl**, we employed a combination of density functional theory (DFT) geometry optimization and machine learning–accelerated Monte Carlo (MC) simulations.

Geometry optimizations

Geometry optimizations of **T.Br-α**, **T.Cl-α**, and **TTBT.Cl** were carried out using the DFT code FHI-aims electronic structure package (version 240920). [10] We employed *tight* species defaults for all elements and a k-point mesh with a reciprocal space sampling density of at least 0.05 x 2π $Å^{-1}$. The self-consistent field (SCF) convergence criterion was set to 10^{-6} eV. For exchange–correlation, we used the Perdew–Burke–Ernzerhof (PBE) generalized gradient approximation [6], augmented by Grimme's D3(BJ) dispersion correction to account for long-range van der Waals interactions. [11] All other parameters were kept at their default values.

Using the above settings, atomic positions were relaxed until the residual forces on each atom were less than 10^{-2} eV $Å^{-1}$. The fully optimized geometries were subsequently used as input for the MC simulations of H_2 and CO_2 adsorption. The final geometries are available in the Supplementary Data deposited at https://doi.org/10.5258/SOTON/D3692.

MC simulations

We used the DFT-optimized geometries of **T.Br-α**, **T.Cl-α**, and **TTBT.Cl** as the hosts, and the DFT-optimized geometries of H_2 and CO_2 as the guest, to perform Metropolis Monte Carlo (MC) sampling and identify binding sites of H_2 in each host. In these simulations, the host geometries were kept fixed. The guest geometries (H_2 and CO_2) were treated as rigid, while adaptive translational and rotational moves were allowed. "Adaptive" here means that the acceptance rate of moves was monitored, and the step size was adjusted accordingly: larger steps were taken if the acceptance rate was too high, and smaller steps if it was too low.

Energies were evaluated using MACE-OMAT-0 (medium), a foundation MACE machine learning interatomic potential (MLIP) [12] trained on the **Open Materials 2024** dataset [13] augmented with a D3 dispersion correction to capture long-range van der Waals interactions. [11] All simulations were carried out using the Atomic Simulation Environment (ASE) toolkit [14] integrated in an in-house branch of mol-CSPy. [15] We performed MC simulations at fixed experimental temperatures: 77 K for H_2 and 298 K for CO_2. Initially, we ran 5,000 MC steps, but since most runs converged within the first 1,000 steps, the total number of steps was reduced to 2,000.

Figure S14 shows an example trajectory for H_2 in **T.Br-α**, where we plot the energy profile along with the changes in centroid position and root-mean-square deviation (RMSD) of the H_2 molecule, both relative to the initial H_2 geometry. This plot clearly showcases the rapid convergence behavior of our adaptive rigid-body MC simulations.

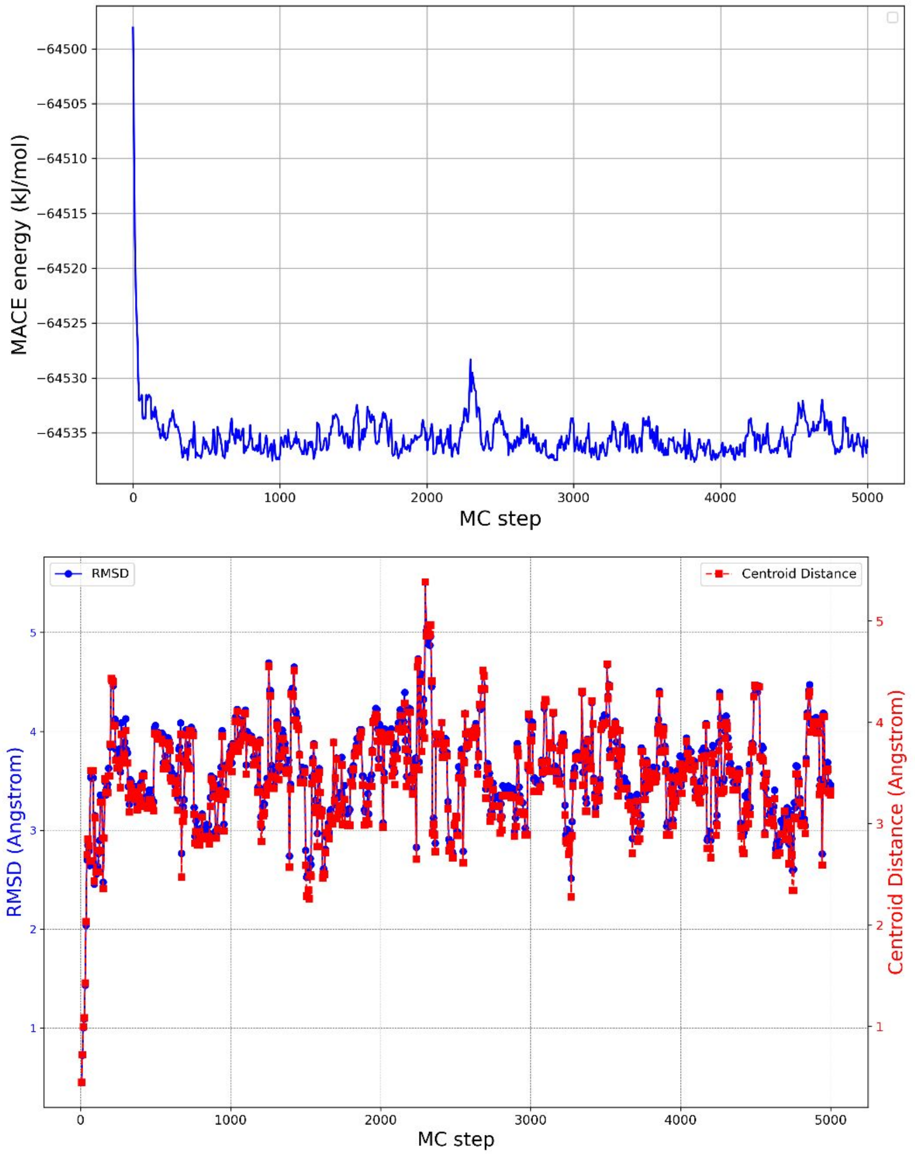

Figure S14. (Top) Energy profile of a MC trajectory of an H_2 molecule within the voids of host **T.Br-α**. (Bottom) Evolution of the H_2 centroid distance (red) and the root-mean-square deviation (RMSD) of atomic positions (blue), both relative to the initial H_2 geometry.

Finding H_2 binding sites

Using the parameters described above, we explored the probable binding sites of H_2 in the voids of **T.Br-α**, **T.Cl-α**, and **TTBT.Cl** by means of MC simulations. For each host system, the initial position of the H_2 molecule was randomized over 20 independent trials. Each trajectory was propagated for 2000 MC steps at the experimental temperature. Convergence was verified by monitoring the energy profile, the RMSD of atomic positions, and the centroid distance of H_2.

The most probable binding sites were then identified by visual inspection of the probability density plots, which represent the likelihood of locating H_2 within the host voids. These probability density distributions were generated in CUBE format (see, for example, Figure S15)

and are provided in the Supporting Data at https://doi.org/10.5258/SOTON/D3692 for each case.

H_2 binding site in T.Br-α and T.Cl-α

In all twenty independent MC trajectories for each of **T.Br-α** and **T.Cl-α**, the H_2 molecule localized in a triangular pocket formed by halogen atoms (Br or Cl, depending on the host) and the triptycene backbone. This pocket corresponds to the most confined and highly polarizable region of the pore; the combination of geometric confinement and the large polarizability of the near halogens produces a deep potential-energy well that stabilizes H_2. The reproducible localization of H_2 across all independent runs indicates a strong thermodynamic preference for this site. Probability density maps of H_2 in **T.Br-α** and **T.Cl-α** are shown in Figures S15 and S16, respectively; these maps are provided in CUBE format in the Supporting Information at https://doi.org/10.5258/SOTON/D3692.

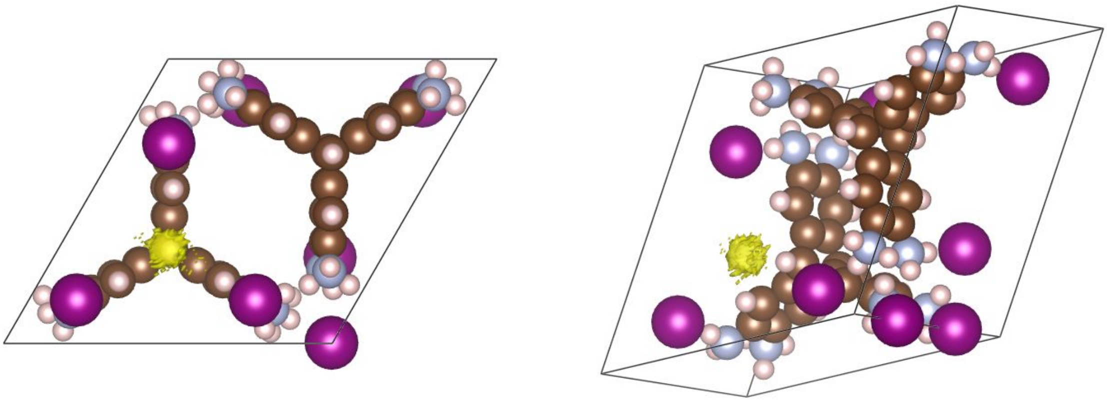

Figure S14. Probability density map of H_2 localization within the voids of **T.Br-α**, shown along the crystallographic *c*-axis (left) and from a side view (right). Carbon, hydrogen, nitrogen, and bromine atoms are depicted in brown, white, blue, and purple, respectively. The yellow isosurface highlights regions of space where H_2 is most likely to be found during the MC trajectory. Graphics are generated using 3D visualization software package VESTA.[17]

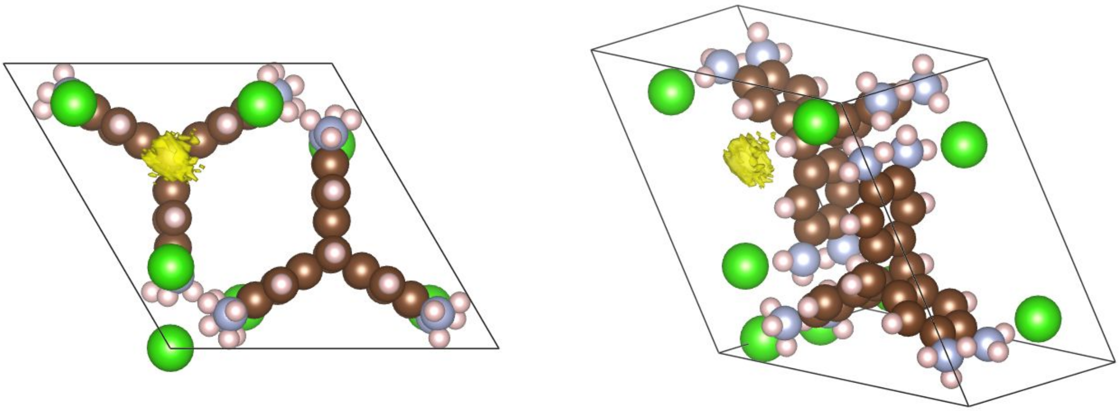

Figure S15. Probability density map of H_2 localization within the voids of **T.Cl-α**, shown along the crystallographic *c*-axis (left) and from a side view (right). Carbon, hydrogen, nitrogen, and chlorine atoms are depicted in brown, white, blue, and green, respectively. H_2 is found in a pocket space similar to where it was previously observed in **T.Br-α.**

H_2 binding site in TTBT.Cl

To rationalize why the new triptycene-based N-MOFs exhibit enhanced H_2 uptake compared to previously reported N-MOF **TTBT.Cl**, it is necessary to identify the preferred binding sites of H_2 in **TTBT.Cl**. The structure of **TTBT.Cl** contains two types of channels, denoted as **A** and **B** in figure 6 of the main text. Each channel A is associated with three channel B's, which have larger diameters. Despite their smaller size, channel A is more polar, suggesting that it may provide a more favorable environment for H_2 adsorption due to stronger dispersion and induction interactions with the framework. However, because the channels are not interconnected, an H_2 molecule can occupy either channel A or one of the channel B's.

Like previous cases, we performed 20 independent MC trajectories with randomized initial positions of H_2 in **TTBT.Cl**. Analysis of these trajectories revealed four distinct binding sites, which are illustrated in Figure S17. Van der Waals (vdW) interactions are the primary driving force for H_2 adsorption in these materials. For **TTBT.Cl**, the stabilization of binding sites 1, 3, and 4 is a result of polarized local environments, where close contacts with polarizable chlorine or nitrogen atoms enhance London dispersion and weak induction interactions. Although binding site 2 is comparatively less polar, geometric confinement within the pore maximizes van der Waals contacts, compensating for the lower polarity and still providing a favorable adsorption site.

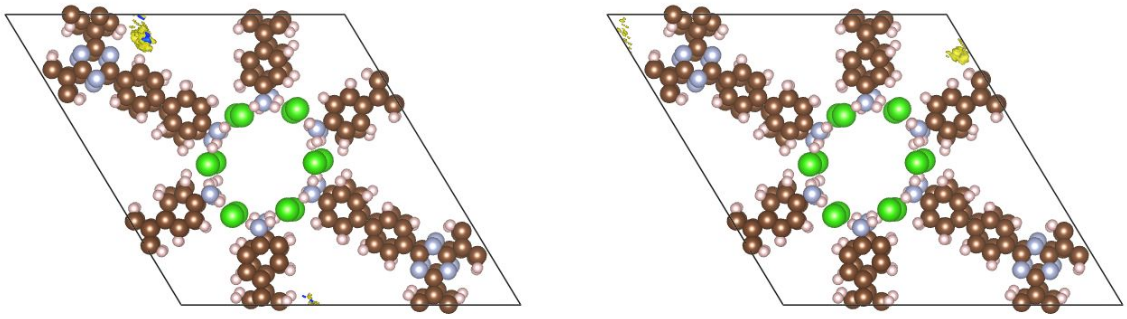

Binding site 1 Binding site 2

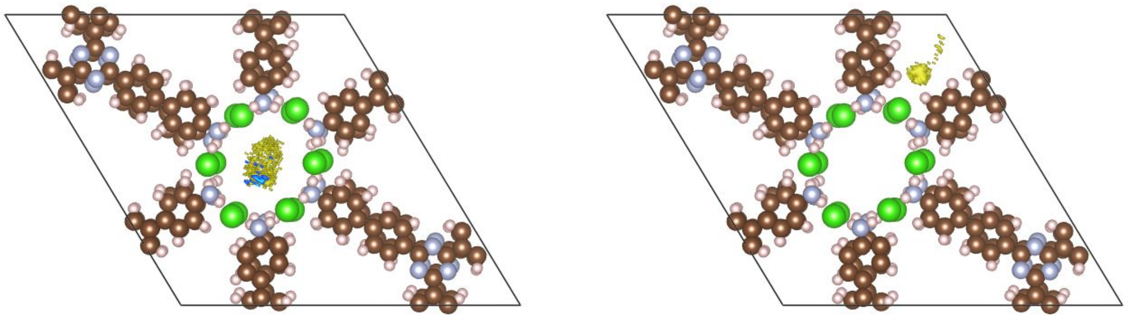

Binding site 3 Binding site 4

Figure S16. Probability density maps of H_2 localization within the voids of **TTBT.Cl**,shown along the crystallographic a-axis for the four possible binding sites predicted by MC simulations. Carbon, hydrogen, nitrogen, and chlorine atoms are depicted in brown, white, blue, and green, respectively.

Binding energies

Although the above MLIP + D3–corrected Monte Carlo simulations provide valuable insight into the probable H_2 binding sites in the three porous host N-MOFs, the accuracy of these predictions requires verification with a DFT approach. To this end, using the FHI-aims code and the computational parameters described above, we performed geometry optimizations of H_2 molecules at all predicted binding sites. During these calculations, the atomic positions of the host framework and the lattice vectors were kept fixed, while only the H_2 molecules were allowed to relax.

The geometry-optimized H_2 molecule deviated only slightly from the Monte Carlo–equilibrated position. Since these DFT optimizations are performed at 0 K, the thermal fluctuations present in the MC simulations are absent. This agreement indicates that the MC results are of reasonable accuracy. The total energies of the isolated host, the gas-phase H_2 molecule, and the combined host–guest system for all studied systems are listed in Table 3.

Table 3. Total energies (PBE+D3) of geometry-optimized hosts, guest molecules, and host–guest systems, along with the calculated binding energies for H_2 adsorption at the binding sites shown in Figures S14–S16.

	Total energy – PBE+D3 (eV)		
T.Br	-631379.89		
T.Cl	-160793.95		
TTBT.Cl	-350653.70		
H_2	-31.75		
		H_2 binding energy (eV)	H_2 binding energy (kJ/mol)
T.Br + 1 H_2	-631411.74	0.0930	8.98
T.Cl+ 1 H_2	-160825.82	0.1149	11.08
TTBT.Cl + 1 H_2 (site 1)	-350685.50	0.0531	5.13
TTBT.Cl + 1 H_2 (site 2)	-350685.49	0.0460	4.44
TTBT.Cl + 1 H_2 (site 3)	-350685.54	0.0928	8.95
TTBT.Cl + 1 H_2 (site 4)	-350685.56	0.1086	10.47

Using these values, the binding energies were calculated as:

$$E_{binding} = E_{host} + E_{guest} - E_{host+guest}$$

The results indicate that the binding energy follows the order: **T.Cl-α (11.08 kJ mol^{-1}) > T.Br-α (8.98 kJ mol^{-1})> TTBT.Cl (average: 7.25 kJ mol^{-1})**. This trend is consistent with the H_2 adsorption capacities reported in Figure 4f of the main text, where **T.Cl-α** exhibits the highest uptake, followed by **T.Br-α**, and then **TTBT.Cl**. These binding energies reflect the combined effects of polarization, van der Waals interactions, and geometric confinement, providing a quantitative confirmation of the experimental adsorption behavior.

The fact that binding energy of H_2 in site 4 (in channel B) of **TTBT.Cl** is larger than site 3 (in channel A) is fully consistent with the dispersion-dominated picture for H_2 adsorption described by Kuc et al., [17] who showed that London dispersion and correlated interactions are the principal stabilizing terms for physisorbed H_2 in MOF-like environments, while classical electrostatic (permanent multipole) contributions are generally secondary; geometric factors and the number/distance of short-range contacts therefore play a decisive role in setting ΔE_{ads}. We calculated Hirshfeld partial charges on the hydrogen atoms in all binding sites, and in every case the magnitude was < 0.02 e. Such negligible charges indicate that the H_2 molecules remain essentially nonpolar upon adsorption.

It is noteworthy to mention that the values quoted above are electronic binding energies computed at the DFT level described above on fixed host geometries; they do not include zero-point energy (ZPE) and thermal/entropic corrections. Inclusion of ZPE and finite-T corrections will change the magnitudes somewhat but is unlikely to invert the qualitative ordering for these deeply binding sites.

Multiple H_2 Adsorption Sites and Loading Capacity

Although the average binding energy trend described above rationalizes the experimentally observed H_2 sorption, it is also instructive to examine how the binding energy evolves as additional H_2 molecules are introduced into the voids of each host system. To this end, we incrementally added H_2 molecules into the pores of **T.Br-α**, **T.Cl-α**, and **TTBT.Cl** randomly and equilibrated the system using the MC methodology described above. H_2 molecules were inserted into one channel until the minimum centroid–centroid distance between adsorbed H_2 molecules reached approximately 3 Å. This distance is significantly smaller than the mean intermolecular separation in the ideal gas (~22 Å) at T = 77 K and p = 1 atm, thereby providing an estimate of the maximum uptake constrained by steric repulsion.

For **T.Br-α** and **T.Cl-α**, H_2 molecules were inserted into a single representative channel, and the results were generalized to the entire system. This approach assumes that adsorption events in different channels are independent of one another. Accordingly, the binding behavior obtained for one channel was taken to be representative of all channels. In the case of **TTBT·Cl**, two distinct types of channels were considered: channel A and channel B. For estimating the overall adsorption capacity, one channel A and three equivalent channel B units were included in the analysis.

For **T.Br-α** and **T.Cl-α**, each one-dimensional channel can accommodate up to five H_2 molecules (15 in total across three channels). The corresponding DFT binding energies for **T.Br-α** and **T.Cl-α**, in the order obtained from the MC simulations, are listed in Table 3. In these cases, two H_2 molecules per channel occupy the pocket formed by the halogen atoms and the triptycene backbone, while the remaining three reside within the main channel. Because the channels in **T.Br-α** and **T.Cl-α** are interconnected, it is highly probable that the first six H_2 molecules exposed to the material preferentially occupy the halogen–triptycene pockets. It is also important to note that in both hosts, one of the channels is blocked by a halogen substituent and therefore inaccessible to H_2 adsorption.

Table 4. Binding energies for a single channel of **T.Br-α** and **T.Cl-α**. Each host contains three equivalent channels; as an approximation, the other channels are assumed to exhibit similar binding energies.

# H_2 molecules	**Binding energy of adding the last H_2 to T.Br-α** (kJ/mol)	**Binding energy of adding the last H_2 to T.Cl-α** (kJ/mol)
1	8.98	11.08
2	9.59	10.75
3	9.70	10.49
4	9.47	10.15
5	9.52	10.31
Average	**9.65**	**10.56**

For **TTBT.Cl**, the adsorption topology is different. Channel A can accommodate up to four H_2 molecules, while each of the three channel B units can hold up to 14 H_2 molecules. The corresponding DFT binding energies in the order obtained from the MC simulations, are listed in Table 5. Considering that **TTBT.Cl** has one channel A and 3 channel B's, in total, this yields a maximum of 46 H_2 molecules (4 + 14 × 3) positioned within the voids of **TTBT.Cl**, based on the 3 Å centroid–centroid separation criterion.

Table 5. Binding energies for channels A and B of **TTBT.Cl** which contains on channel A and three equivalent channel B's; as an approximation, the other channel B's are assumed to exhibit similar binding energies.

# H_2 molecules	**Binding energy of adding the last H_2 to channel B** (kJ/mol)	**Binding energy of adding the last H_2 to channel A** (kJ/mol)
1	10.46	8.95
2	7.86	9.20
3	8.72	9.16
4	7.87	9.37
5	8.49	-
6	8.05	-
7	8.00	-
8	8.11	-
9	8.41	-
10	8.32	-
11	8.26	-
12	8.07	-
13	8.10	-
14	8.14	-
Average	**8.35**	**9.17**

The corresponding MC trajectories and DFT-relaxed geometries (with the host framework kept fixed) are available in the Supplementary Data deposited at https://doi.org/10.5258/SOTON/D3692.

Average Binding Energies and Comparison to Experiment

By averaging over the binding energies reported in Tables 4 and 5, we estimated the mean stabilization energy per H_2 molecule in each host system as follows: **T.Br-α**, 9.65 kJ mol^{-1}; **T.Cl-α**, 10.56 kJ mol^{-1}; and **TTBT.Cl**, 8.42 kJ mol^{-1} $(14 \times 3 \times \ 8.35 \ + \ 9.17 \times \ 4) \,/\, 46$.

Previously, we showed that based on the binding energies of individual H_2 molecules at the preferred adsorption sites, the relative order of affinity is **T.Cl-α > T.Br-α > TTBT.Cl**. The same ordering emerges when considering the collective adsorption of multiple H_2 molecules using the averaging approach described above. Thus, the experimentally observed H_2 uptake trends are fully consistent with the combined MC and DFT simulations.

It should be emphasized, however, that several effects are not captured in these calculations. These include finite-temperature contributions, the approximate treatment of dispersion via the geometrical D3 correction, dynamical fluctuations of the adsorbed molecules, kinetic limitations in the experiment, and lattice defects in real materials. Incorporating such effects would further refine the accuracy of the predicted adsorption energetics.

CO_2 Adsorption Sites in T.Br-α, T.Cl-α, and TTBT.Cl

Using the combined MC + DFT methodology described above, we identified the most probable binding sites for CO_2 within the pores of **T.Br-α**, **T.Cl-α**, and **TTBT.Cl**, as illustrated in Figures S18–S20, respectively.

In **T.Br-α** and **T.Cl-α**, CO_2 experiences significant steric hindrance in approaching the pockets formed by the halogen atoms and the triptycene backbone. Consequently, the preferred binding sites for CO_2 in these hosts differ from those identified for H_2, although CO_2 still preferred strongly to bind near halogen atoms. In contrast, for **TTBT.Cl**, the binding sites for CO_2 are like those for H_2, as the binding sites are sufficiently open to accommodate CO_2 molecules without significant steric constraints.

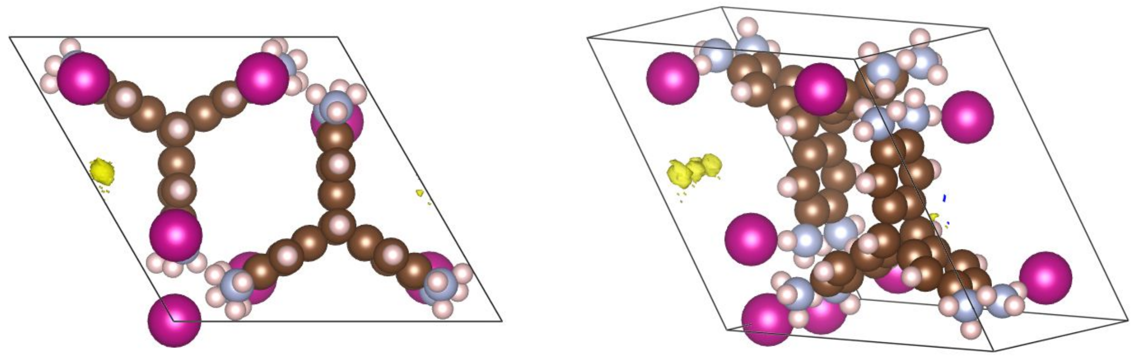

Figure S18. Probability density map of CO_2 localization within the voids of **T.Br-α**, shown along the crystallographic *c*-axis (left) and from a side view (right). Carbon, hydrogen, nitrogen, and bromine atoms are depicted in brown, white, blue, and purple, respectively. The yellow isosurface highlights regions of space where CO_2 is most likely to be found during the MC trajectory.

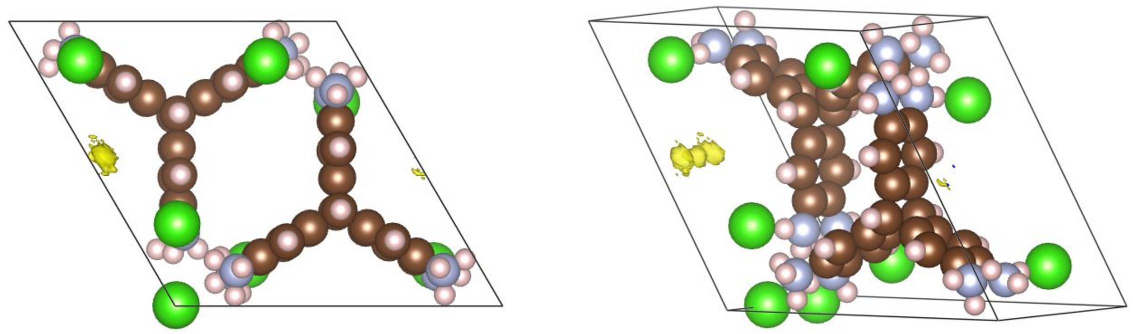

Figure S19. Probability density map of CO_2 localization within the voids of **T.Cl-α**, shown along the crystallographic *c*-axis (left) and from a side view (right). Carbon, hydrogen, nitrogen, and chlorine atoms are depicted in brown, white, blue, and green, respectively.

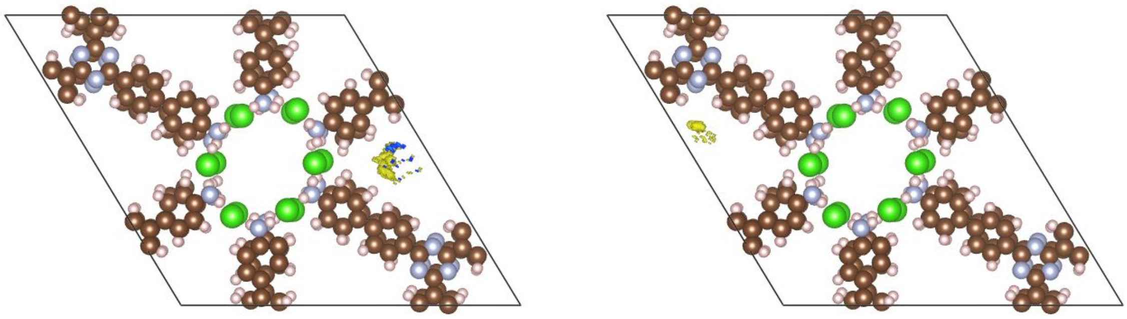

Binding site 1 Binding site 2

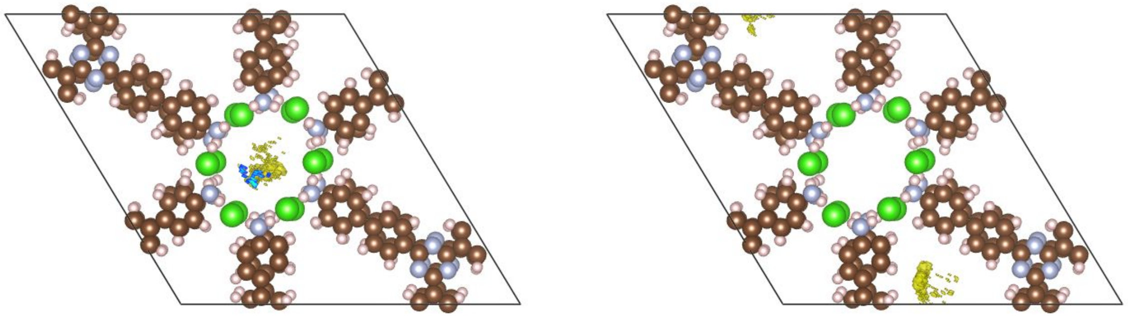

Binding site 3 Binding site 4

Figure S20. Probability density maps of CO_2 localization within the voids of **TTBT.Cl**,shown along the crystallographic a-axis for the four possible binding sites predicted by MC simulations. Carbon, hydrogen, nitrogen, and chlorine atoms are depicted in brown, white, blue, and green, respectively.

Analogous to the H_2 case, geometry optimizations of CO_2 molecules at all predicted binding sites were performed using FHI-aims DFT code with the same computational parameters. During these calculations, only the CO_2 molecules were allowed to relax, while the atomic positions of the host framework and the lattice vectors were kept fixed. The deviations of CO_2 geometries from their Monte Carlo–equilibrated positions were minimal, re-confirming the reliability and accuracy of the MLIP-powered MC results. The total energies of the isolated host, gas-phase CO_2, and the combined host–guest systems for all studied cases are summarized in Table 6.

Table 6. Total energies (PBE+D3) of geometry-optimized hosts, guest molecules, and host–guest systems, along with the calculated binding energies for CO_2 adsorption at the binding sites shown in Figures S18–S20.

	**Total energy – PBE+D3 (eV)**		
T.Br	-631379.89		
T.Cl	-160793.95		
TTBT.Cl	-350653.70		
CO_2	-5134.50		
		**CO_2 binding energy (eV)**	**CO_2 binding energy (kJ/mol)**
T.Br + 1 CO_2	-636514.71	0.3140	30.30
T.Cl+ 1 CO_2	-165928.77	0.3092	29.84

TTBT.Cl + 1 CO_2 (site 1)	-355788.42	0.2259	21.79
TTBT.Cl + 1 CO_2 (site 2)	-355788.39	0.1939	18.71
TTBT.Cl + 1 CO_2 (site 3)	-355788.51	0.3077	29.69
TTBT.Cl + 1 CO_2 (site 4)	-355788.42	0.2258	21.78

The results in Table 5 indicate that the binding energy follows the order: **T.Br-α (30.30 kJ mol^{-1})> TTBT.Cl (average: 22.99 kJ mol^{-1})**. This trend is consistent with the adsorption capacities reported in Figure 4e of the main text, where **T.Br-α** exhibits a higher CO_2 uptake than **TTBT·Cl**. The stronger binding in **T.Br-α** can be attributed to a combination of factors: van der Waals interactions between CO_2 and the pore walls, and the geometric confinement imposed by the channel topology and enhanced polarization effects due to more polar channels. Together, these effects are reflected in an improved binding energy which provides a quantitative explanation for the experimentally observed differences in CO_2 uptake.

CSP of Triptycene Halides

CSP was performed using the Global Lattice Energy Explorer program, which employs low-discrepancy, quasi-random sampling of crystal packing variables to generate a uniform sampling of the lattice energy surface. The cation geometry was kept rigid throughout the entire CSP process. Trial crystal structures were generated across 10 space groups, and their lattice energies were minimised until a target number of valid crystal structures was met (Table 6). Rigid-molecule lattice energy optimisations were performed using the DMACRYS software. Lattice energies were calculated using an anisotropic atom–atom energy model based on a revised version of the Williams 99 force-field, combined with atom-centred multipoles calculated from a distributed multipole analysis of the PBE0/6-311 G** density. Multipoles up to hexadecapole on each atom were included, and the polarizable continuum model was applied to the distributed multipole analysis to further improve the electrostatic model, using a dielectric constant of 3.0. Bromide parameters were the same as those used in our previous study. Duplicate crystal structures were removed from the final CSP landscape by calculating the similarities of simulated PXRD patterns. Channel dimensionalities were calculated using the Zeo++ software package and a probe radius of 1.45 Å.

Table 7. List of space groups that were sampled, and number of generated structures per space group, in the CSP study.

Space groups	Number of valid structures to generate
$P1$, $P\bar{1}$, $P2_12_12_1$	10,000 each
$Pna2_1$, $P2_1$, $C2$, Cc	20,000 each
$P2_1/c$, $C2/c$, $R\bar{3}$,	50,000 each

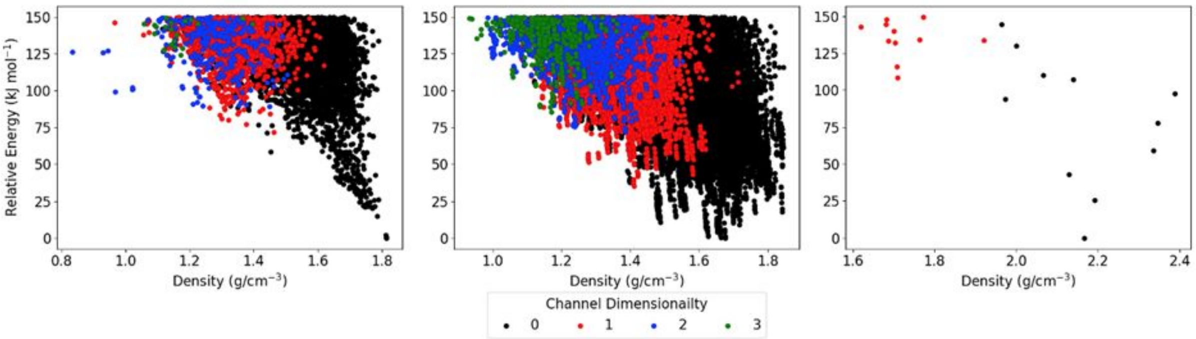

Figure S21. CSP Landscapes of **T.Br** when using a 3+ TT cation (left), 4+ TT cation (middle) and 6+ TT cation (right). The relative energy axis has been truncated at 150 kJ mol^{-1}. Points are coloured by their calculated pore dimensionality. None of these salt combinations is predicted to show pore structures as the global minimum crystal packing, but the 4+ case (middle plot) shows 1-D porous structures (red points) that are around 30 kJ mol^{-1} in energy above the non-porous global minimum predicted structures (black points). This is within the range that might be accessible via solvent stabilization. Hence, while these CSP calculations cannot account for the observed anion disorder, and therefore do not reproduce the observed experimental structure, they do suggest that the observed 4+ TT salt is more likely to form 1-D porous structures than the alternative 3+ or 6+ compositions.

References

1 Feike, M. *et al.* Broadband multiple-quantum NMR spectroscopy. *J Magn Reson Ser A* **122**, 214-221 (1996).

2 Cordova, M., Balodis, M., Simoes de Almeida, B., Ceriotti, M. & Emsley, L. Bayesian probabilistic assignment of chemical shifts in organic solids. *Sci Adv* **7**, eabk2341 (2021).

3 Giannozzi, P. *et al.* Advanced capabilities for materials modelling with QUANTUM ESPRESSO. *Journal of Physics-Condensed Matter* **29** (2017). 4 Giannozzi, P. *et al.*

QUANTUM ESPRESSO: a modular and open-source software project for quantum simulations of materials. *Journal of Physics-Condensed Matter* **21** (2009).
4 Perdew, J. P. in *AIP Conference Proceedings* 1-20 (2001).
5 Perdew, J. P., Burke, K. & Ernzerhof, M. Generalized Gradient Approximation Made Simple. *Phys Rev Lett* **77**, 3865-3868 (1996).
6 Grimme, S. Semiempirical GGA-type density functional constructed with a long-range dispersion correction. *J Comput Chem* **27**, 1787-1799 (2006).
7 Grimme, S., Antony, J., Ehrlich, S. & Krieg, H. A consistent and accurate ab initio parametrization of density functional dispersion correction (DFT-D) for the 94 elements H-Pu. *J Chem Phys* **132**, 154104 (2010).
8 Rabbani, M. G.; Reich, T. E.; Kassab, R. M.; Jackson, K. T.; El-Kaderi, H. M. High CO_2 uptake and selectivity by triptycene-derived benzimidazole-linked polymers. *Chemical Communications* **2012**, *48* (8), 1141-1143
10 Blum, V., Gehrke, R., Hanke, F., Havu, P., Havu, V., Ren, X., ... & Scheffler, M. (2009). Ab initio molecular simulations with numeric atom-centered orbitals. *Computer Physics Communications*, 180(11), 2175-2196.
11 Grimme, S., Ehrlich, S. & Goerigk, L. Effect of the damping function in dispersion corrected density functional theory. *J. Comput. Chem.* 32, 1456–1465 (2011).
12 Batatia, I., Kovacs, D. P., Simm, G., Ortner, C., & Csányi, G. (2022). MACE: Higher order equivariant message passing neural networks for fast and accurate force fields. *Advances in Neural Information Processing Systems*, 35, 11423-11436.
13 Barroso-Luque, L., Shuaibi, M., Fu, X., Wood, B. M., Dzamba, M., Gao, M., ... & Ulissi, Z. W. (2024). Open materials 2024 (omat24) inorganic materials dataset and models. arXiv preprint arXiv:2410.12771.
14 Larsen, Ask Hjorth, et al. The atomic simulation environment—a Python library for working with atoms. *Journal of Physics: Condensed Matter* 29.27 (2017): 273002.
15 https://gitlab.com/mol-cspy/mol-cspy
16 Momma, Koichi, and Fujio Izumi. "VESTA 3 for three-dimensional visualization of crystal, volumetric and morphology data." *Applied Crystallography* 44.6 (2011): 1272-1276.
17 Kuc, A., Heine, T., Seifert, G., & Duarte, H. A. (2008). H_2 Adsorption in Metal-Organic Frameworks: Dispersion or Electrostatic Interactions?. *Chemistry–A European Journal*, (22), 6597-6600.
